# Interferon-γ inducible factor 16 (IFI16) restricts adeno-associated virus type 2 (AAV2) transduction in an immune-modulatory independent way

**DOI:** 10.1128/jvi.00110-24

**Published:** 2024-06-05

**Authors:** Sereina O. Sutter, Kurt Tobler, Michael Seyffert, Anouk Lkharrazi, Joël Zöllig, Elisabeth M. Schraner, Bernd Vogt, Hildegard Büning, Cornel Fraefel

**Affiliations:** 1 Institute of Virology, University of Zurich, Zurich, Switzerland; 2 Institute of Experimental Hematology, Hannover Medical School, Hannover, Germany; International Centre for Genetic Engineering and Biotechnology, Trieste, Italy

**Keywords:** adeno-associated virus, global gene expression analysis, RNA sequencing, vector-mediated cell transduction, innate immune responses, interferon-γ inducible factor 16

## Abstract

We determined the transcription profile of adeno-associated virus type 2 (AAV2)-infected primary human fibroblasts. Subsequent analysis revealed that cells respond to AAV infection through changes in several significantly affected pathways, including cell cycle regulation, chromatin modulation, and innate immune responses. Various assays were performed to validate selected differentially expressed genes and to confirm not only the quality but also the robustness of the raw data. One of the genes upregulated in AAV2-infected cells was interferon-γ inducible factor 16 (IFI16). IFI16 is known as a multifunctional cytosolic and nuclear innate immune sensor for double-stranded as well as single-stranded DNA, exerting its effects through various mechanisms, such as interferon response, epigenetic modifications, or transcriptional regulation. IFI16 thereby constitutes a restriction factor for many different viruses among them, as shown here, AAV2 and thereof derived vectors. Indeed, the post-transcriptional silencing of *IFI16* significantly increased AAV2 transduction efficiency, independent of the structure of the virus/vector genome. We also show that IFI16 exerts its inhibitory effect on AAV2 transduction in an immune-modulatory independent way by interfering with Sp1-dependent transactivation of wild-type AAV2 and AAV2 vector promoters.

**IMPORTANCE:**

Adeno-associated virus (AAV) vectors are among the most frequently used viral vectors for gene therapy. The lack of pathogenicity of the parental virus, the long-term persistence as episomes in non-proliferating cells, and the availability of a variety of AAV serotypes differing in their cellular tropism are advantageous features of this biological nanoparticle. To deepen our understanding of virus-host interactions, especially in terms of antiviral responses, we present here the first transcriptome analysis of AAV serotype 2 (AAV2)-infected human primary fibroblasts. Our findings indicate that interferon-γ inducible factor 16 acts as an antiviral factor in AAV2 infection and AAV2 vector-mediated cell transduction in an immune-modulatory independent way by interrupting the Sp1-dependent gene expression from viral or vector genomes.

## INTRODUCTION

Adeno-associated virus serotype 2 (AAV2) is a small, non-pathogenic, helper virus-dependent parvovirus with a single-stranded (ss) DNA genome of approximately 4.7 kb, which has attracted interest as a basis for one of the most frequently applied vector system in human gene therapy (1). In the absence of a helper virus, such as herpes simplex virus type 1 (HSV-1), the AAV2 genome can persist as an episome in the nucleus or integrate site-preferentially into the adeno-associated virus pre-integration site (AAVS1) on human chromosome 19 (2, 3). Co-infection with the helper virus promotes lytic replication and production of progeny virus (4). The AAV2 genome contains two large open reading frames (ORFs), which are flanked on either side by 145 nt long inverted terminal repeats. *Rep* gene expression from two different promoters (p5 and p19) results in the synthesis of four non-structural Rep proteins (Rep78, Rep68, Rep52, and Rep40) by employing alternative splice sites at map positions 42 and 46 (5, 6). The activity of the p5 and p19 promoters is regulated by the Rep binding site, allowing Rep to act as either a repressor or transactivator (7). In the absence of a helper virus, only small amounts of Rep are produced, which nevertheless can repress any further transcription.

The icosahedral AAV2 capsid is built by the three structural proteins VP1, VP2, and VP3, which are encoded by the *cap* gene. Two additional proteins, the assembly-activating protein and the membraneassociated accessory protein, are encoded by the *cap* gene via nested alternative ORFs (8, 9).

RNA sequencing (RNA-seq) is a technology that uses the commitment of next-generation sequencing (NGS), also known as deep sequencing, to identify transcripts and their quantity in cells at a given time point (10). The development of NGS with its high base coverage and sample throughput facilitates the sequencing of transcripts in cells and allows to study alternative spliced transcripts, changes in gene expression, and cellular pathway alterations during infection (11). Moreover, RNA-seq facilitates a closer look at different types of RNA (e.g., mRNA, sRNA, tRNA, and miRNA), ribosome profiling, and the total RNA content of a cell (12) by overcoming the limited coverage and inability to detect rare transcript variants. Using this approach, we provide here a genome-wide expression profile of AAV2-infected cultured fibroblasts, unveiling virus-host interactions. Transcript mapping, followed by an overall expression counting resulted in 44,175 annotations. The deeper analysis of differentially expressed genes between AAV2- and mock-infected cells revealed 1,929 distinct (*P* < 0.01, number of reads ≥ 40) regulated genes, of which 92.78% were protein coding, including among others the interferon-inducible p200-family protein IFI16. IFI16 is assumed to be an innate immune sensor for cytosolic and nuclear double-stranded (ds), as well as ssDNA (13). IFI16 has been shown to be a restriction factor for many different viruses through various mechanisms, including interferon response, transcriptional regulation, and epigenetic modifications. For example, human cytomegalovirus (HCMV) replication was shown to be significantly enhanced due to IFI16-mediated blockage of Sp1-dependent transcription of UL54 (14). Moreover, IFI16 can also restrict HSV-1 replication by repressing HSV-1 gene expression, independently of its roles in the immune response (13), via global histone modifications by decreasing the markers for active chromatin and increasing the markers for repressive chromatin on cellular and viral genes (15, 16). IFI16, however, inhibits not only various DNA viruses such as HCMV, HSV-1, or human papillomavirus 18 (17) but also shares properties of known anti-retroviral restriction factors (18) and blocks human immunodeficiency virus type 1 (HIV-1) by binding and inhibiting the host transcription factor Sp1 that drives viral gene expression (19), similar to HCMV.

AAV2 and AAV2 vectors deliver ssDNA or dsDNA or induce the formation of ssDNA, dsDNA, and circular dsDNA products and may, therefore, provoke an IFI16-triggered reaction. Consequently, we aimed to address the question of whether IFI16 influences AAV2 infection and vector-mediated cell transduction, respectively.

## RESULTS

### Total RNA-seq reveals 1,929 d**ifferentially expressed genes in AAV2-infected versus mock-infected normal human fibroblasts**

Structural cells, such as fibroblasts, are found in literally every tissue, making them susceptible to a variety of AAV serotypes and thereof derived vectors. Although their molecular signature is not maintained between organs (20), tissue-resident fibroblasts were shown to play a key role in the suppression or activation of immune responses [reviewed in reference (21)]. To assess the global gene expression profile of AAV2 and mock-infected normal human fibroblast (NHF) cells, RNA-seq was performed on total RNA isolated 24 hours post-infection (hpi). Transcript mapping to the human assembly and gene annotation from Ensembl, extended by the AAV2 sequence, followed by an overall expression counting (see Materials and Methods) resulted in 44,175 annotations. The analysis revealed 1,929 differentially expressed (DE) genes (*P* < 0.01, number of reads ≥ 40) between AAV2- and mock-infected cells, of which 92.78% were protein coding. A small portion of the restricted annotation resulted in pseudo genes and anti-sense RNA (Table 1).

**TABLE 1:** Summary of the gene annotation and numbers of differentially expressed genes at 24 hpi

Type	Annotation	*P* < 0.01, number of reads ≥ 40
Protein coding	19,934	1,787
Small RNA	1,286	2
rRNA	102	0
Pseudogene	9,948	70
lincRNA	5,862	13
Antisense	4,701	41
Other	2,339	13
AAV specific	3	3
Total	44,175	1,929

### The biological process ontology of AAV2-infected cells

**Fig 1:**
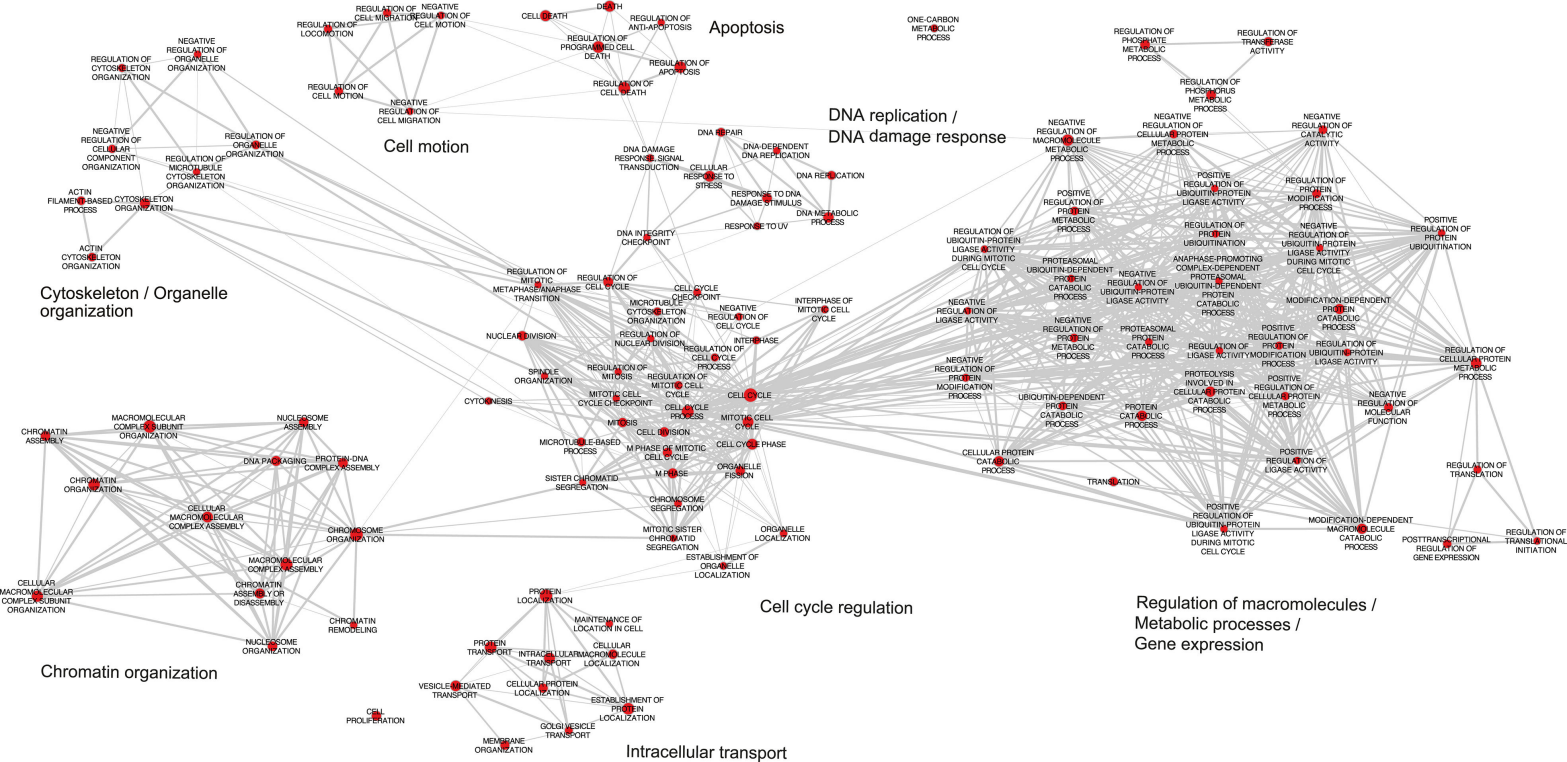
Enrichment map of DAVID GO terms. Analysis of 1,930 differentially expressed genes (*P* < 0.01, number of reads > 40) by DAVID was used to create an enrichment map of the GO terms using Cytoscape. Nodes (red) represent the individual GO terms, while their size corresponds to the number of genes included. Edges (gray lines) represent mutual overlaps, and the thickness reflects the number of overlaps. The most affected biological processes are summarized as keywords.

To further explore the 1,929 differentially regulated genes, a Gene Ontology (GO) term biological process (BP) analysis was performed. The GO term BP was identified by DAVID (22, 23) and graphically visualized as an enrichment map using Cytoscape. Eight distinct clusters of biological processes that differed between AAV2- and mock-infected cells became evident (Fig. 1; Tables S1 and S2). The cluster termed “regulation of macromolecule/metabolic processes/gene expression” was heavily represented with 38 nodes, followed by the cluster “cell cycle regulation” with 30 nodes. Considering that the size of the nodes within the clusters corresponded to the number of genes included, some GO terms were represented by more genes than others. Taking this into account, the clusters “cell cycle regulation,” “chromatin organization,” “DNA replication/damage response,” and “apoptosis” were highly represented. This suggested that AAV2 can modulate crossroads of host gene expression relevant for cell cycle regulation, chromatin modulation, DNA-damage response, and apoptosis. To further analyze and visualize the DE genes within the cell cycle and chromatin organization clusters, unsupervised hierarchical clustering of the expression levels from the 50 most differently expressed genes of those GO terms was generated. The DE genes were ordered according to their absolute difference in expression with the most differentially regulated genes at the top (see Fig. S1). The highest fold difference in the chromatin organization cluster was 5.4 for *HIST1H2AJ*, with the smallest difference of 2.2 for *MCM2*, whereby the majority of differentially expressed genes belonged to the histone cluster 1 genes. In the case of the cell cycle regulation cluster, *CDC45* and *UBE2I* showed a maximum/minimum fold difference of 2.8 and 1.8, respectively. Overall, the differential expression profile of the cell cycle showed a broader range of genes included, compared to the uniform pattern of the chromatin organization group, consisting mostly of histone cluster 1 genes.

### Identification of the number of genes with a fold change of ≥1.5

**Fig 2:**
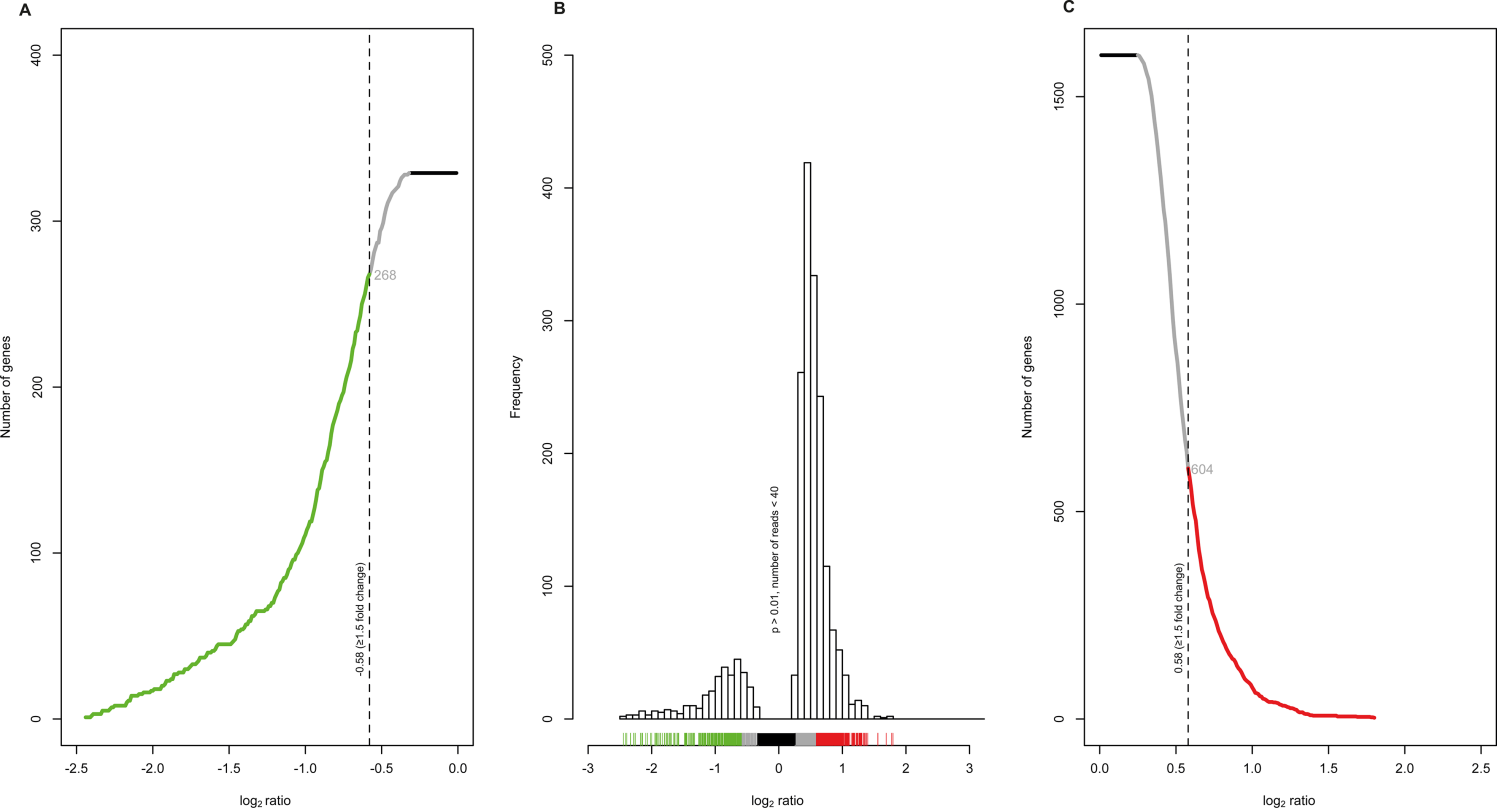
Identification of the number of genes with a fold change of ≥1.5. The number of genes with a log_2_ ratio of mean read abundance of transcripts (AAV2-infected NHF cells to mock-infected NHF cells) between (**A**) −0.01 and −2.5 or (**C**) 0.01 and 2.5, respectively, and a significance threshold of *P* < 0.01. The dashed vertical line in (**A**) −0.58 and (**C**) 0.58 indicates a fold change of 1.5. The number of genes representing a fold change ≥ 1.5 is numerically indicated in gray (872 genes in total). The number of genes (**A**) below a log_2_ ratio of −0.58 is illustrated in green, whereas those (**C**) with a log_2_ ratio greater than 0.58 are depicted in red. The same color code was used for (**B**) the distribution frequency. The black bar in panel **B** indicates those log_2_ values, which were directly excluded.

The remaining 1,929 DE genes were further screened with more restrictive criteria. Specifically, genes with a fold change (FC) higher than 1.5, which is equivalent to a log_2_ ratio of the mean transcript abundance of AAV2 to mock-infected cells of > |0.58|, were selected. Overall, 268 (Fig. 2A) and 604 (Fig. 2C) cellular genes were down- or upregulated, respectively. Figure 2B, however, illustrates the entire distribution frequency of the log_2_ ratio of the mean read abundance of the transcripts of AAV2 to mock-infected cells. Overall, 872 genes were defined as differentially expressed at least 1.5-fold in AAV2-infected cells compared to mock-infected cells.

To compare the transcript read abundance of the 872 genes between AAV2-infected and mock-infected cells, an unsupervised clustered heat map was generated (see Fig. S2 ). The read abundance of transcripts (log_2_) ranged from roughly 0.54 (M2) to approximately 16 (A3). Furthermore, the heat map showed that the three samples from AAV2-infected cells were homogeneous and clearly differed from the mock-infected cells. However, the mock-infected M2 sample did not correlate with M1 and M3.

### Functional classification of the transcriptome following AAV2 infection

**Fig 3:**
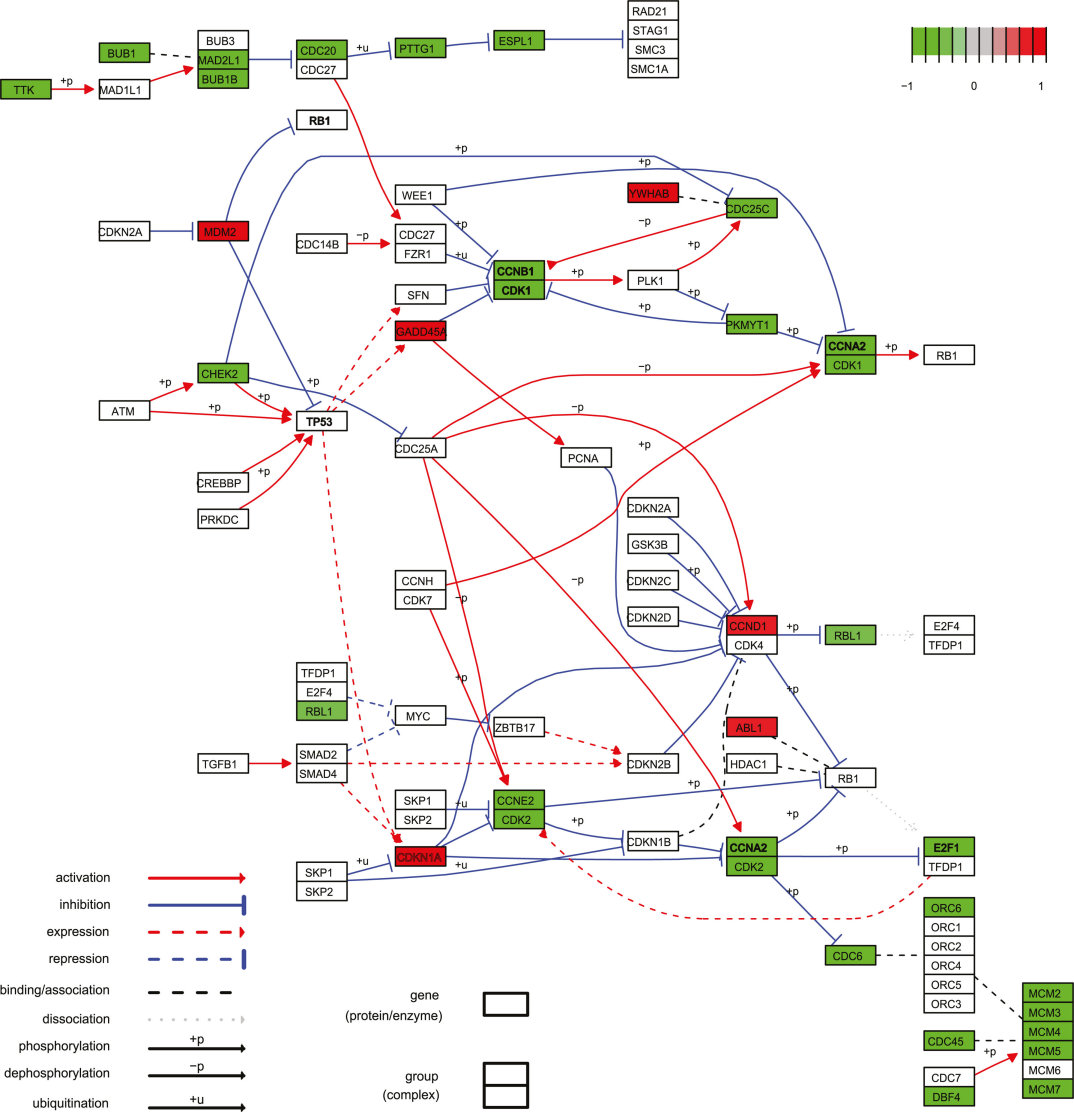
KEGG pathway analysis of the cell cycle allowed the identification of differentially expressed genes in AAV2 and mock-infected cells. Upregulated genes are color coded in red, while downregulated genes are depicted in green (FC ≥ 1.5, *P* < 0.01, number of reads > 40). Symbol legend is shown in the KEGG pathway analysis.

To further analyze the RNA-seq data, the list of 872 differentially expressed genes with an FC ≥ 1.5 was projected onto the KEGG pathways (24). The KEGG analysis revealed that the majority of the genes involved in cell cycle regulation were downregulated upon AAV2 infection (Fig. 3). Several cyclins as well as their binding partners, the cyclin-dependent kinases (CDKs), which are relevant for the progression of the cell into the S-phase and further transition into the G2-phase, were downregulated. These downregulations also influence the expression state of several transcription factors (E2Fs), spindle checkpoint proteins (MAD2 and BUBR1), and replication-relevant proteins (MCMs and ORC). Moreover, some anaphase-promoting complex proteins (ESP1 and PTTG) were also downregulated. Some of the upregulated genes negatively regulate those genes that were downregulated, such as the growth arrest and DNA-damage-inducible protein (GADD45), which negatively affects the binding of CDK1 to cyclin B1 (25, 26), and the CDK inhibitory protein (CIP1), also known as CDKN1A or p21. Others, like the 14-3-3 σ protein (encoded by *YWHAB*), D-type cyclins, and the oncoprotein MDM2 have a direct influence on the cell cycle progression. The upregulated abelson tyrosine-protein kinase 1 (ABL1) is involved in DNA-damage response and apoptosis, as well as in the phosphorylation of several cell cycle-relevant proteins, such as the retinoblastoma (RB) protein (27) or proteasome subunit alpha type-7 (PSMA7), thereby influencing their activation state and protein interactions.

### Validation of the transcriptome data *in vitro*

**Fig 4:**
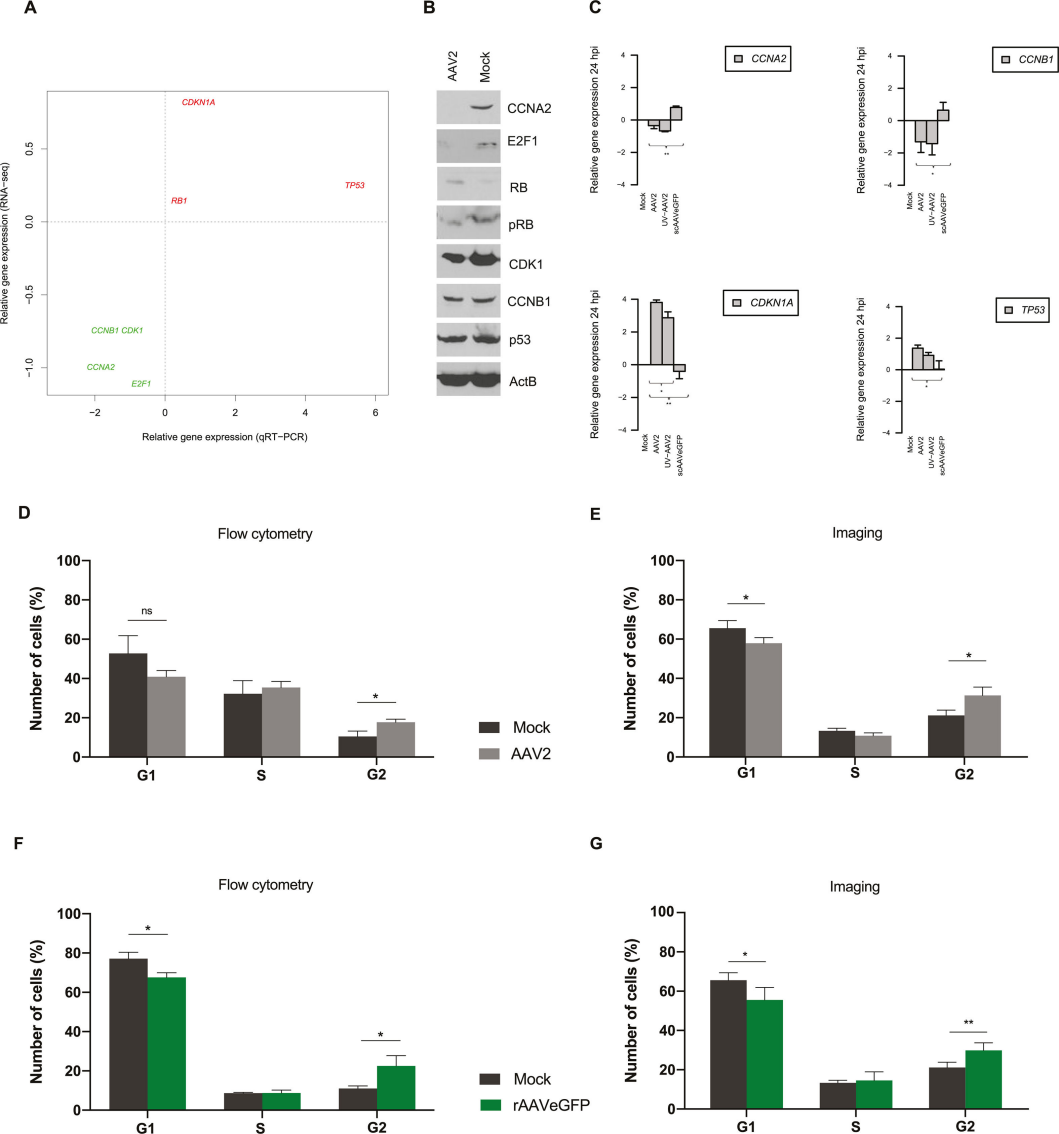
Validation of the transcriptome data *in vitro.* (A) Comparison of relative gene expression profile of selected genes from AAV2-infected versus mock-infected NHF cells at 24 hpi by RT-qPCR and RNA-seq. Upregulated genes are color coded in red, while downregulated genes are depicted in green. All data generated by RT-qPCR were normalized against the mean of two housekeeping genes *GAPDH* and *SDHA*. Data shown are the means of triplicates. (B) Total cell lysates of mock-infected (Mock) and AAV2-infected cells prepared at 48 hpi were subjected to Western blot analysis. The β-actin stain was used as a loading control. (C) Relative gene expression profiles of selected genes in NHF cells infected with AAV2, UV-irradiated AAV2 (UV-AAV2), or self-complementary AAV2 (scAAVeGFP) at 24 hpi. All data generated by RT-qPCR were normalized against the housekeeping gene *SDHA*. Graphs show mean and standard deviation (SD) from triplicate experiments. The unpaired Student’s *t*-test was used to determine the significance (*P ≤ 0.05, **P ≤ 0.01, ***P ≤ 0.001, and ****P ≤ 0.0001) of the differences between the expression profiles of AAV2-, UV-AAV2-, and scAAVeGFP-infected cells (multiplicity of infection [MOI], 500). UV inactivation was assessed on protein level using an anti-Rep antibody (data not shown). Cell cycle profile of AAV2- or rAAVeGFP (MOI 5,000)-infected NHF cells at 24 hpi assessed either by (D and F) propidium iodide staining and flow cytometry (10,000 cells per sample) or by (E and G) 4′,6-diamidino-2-phenylindole staining and confocal laser scanning microscopy (100 cells per sample). Graphs show mean and SD of the percentage of cells in each cell cycle phase. *P*-values were calculated using an unpaired Student’s *t*-test (*P ≤ 0.05, **P ≤ 0.01, ***P ≤ 0.001, and ****P ≤ 0.0001).

In order to assess the *in silico*-affected biological process, specifically cell cycle regulation, transcript and protein levels of selected genes were evaluated by quantitative reverse transcription PCR (RT-qPCR) and Western blot (WB) analysis, respectively (Fig. 4A and B).

The RT-qPCR data were normalized to the mean value of the transcriptional activity of two housekeeping genes (*GAPDH* and *SDHA*) since their expression profile was only marginally affected (SDHA; log_2_ = 0.2, *P* = 0.3; GAPDH; log_2_ = 0.2, *P* = 0.04) upon AAV2 infection. The resulting ∆∆C_t_ values of each gene of interest were plotted against their corresponding log_2_ ratio of the mean transcript abundance of AAV2 to mock-infected cells from the RNA-seq data (Fig. 4A). The expression levels of *CCNA2*, *CCNB1*, *CDK1,* and *RB1* correlated well between the RT-qPCR and the RNA-seq data (*R*^2^ = 0.897), although for *E2F1*, *CDKN1A,* and *TP53,* a moderate difference of the RT-qPCR data to the RNA-seq data was observed. Next, Western blot analysis of total cell lysates at 48 hpi was performed to evaluate the protein expression levels of selected genes recognized by RNA-seq to be differentially expressed between AAV2-infected and mock-infected cells. The 48-h time point of AAV2 infection was chosen because the differential regulation on protein level might lag behind that observed on the level of transcription. The immunoblotting, using specific antibodies for each protein of interest (Fig. 4B), showed a strong reduction of E2F1, CCNA2, and CDK1 on protein level in AAV2-infected cells. No obvious change after the AAV2 infection was detected for total CCNB1 levels, whereas a moderate increase in total RB levels was observed and almost no change in total p53 levels, showing that the RNA-seq and total protein levels accorded well. To further assess the downregulation of E2F1 in AAV2-infected cells, the level of activated RB protein was determined by Western blot analysis using a phospho-specific antibody. Since E2F1 is kept in an inactive state through its interaction with the hypophosphorylated form of RB, enhanced activity of cyclin E and A would normally lead to the phosphorylation of RB, resulting in a reduced affinity of RB for E2F1, thereby causing its release and activation, leading to enhanced expression of cyclin A and other S-phase genes. This protein interplay between cyclin A, E2F1, and the activation state of RB can be readily observed in Fig. 4B (mock-infected cells). In contrast, a reduced level of activated RB, E2F1, and CCNA2 and an elevated level of hypophosphorylated RB in the AAV2-infected cells were observed. Overall, the transcript and protein expression levels of the selected genes were in agreement with the transcription levels and connote a differential regulation of genes relevant for cell cycle progression upon AAV2 infection.

Next, we addressed the question of whether the single-stranded AAV2 DNA *per se* or low-level AAV2 *rep* gene expression, as *rep* transcripts were indeed identified by RNA-seq, caused the changes in host gene expression. To this end, NHF cells were infected with AAV2, UV-irradiated AAV2 (UV-AAV2), or an AAV2 vector, delivering a vector genome in the self-complementary (sc) genome configuration expressing an enhanced green fluorescent protein (eGFP; scAAVeGFP). The expression of four genes (*CCNA2*, *CCNB1*, *CDKN1A,* and *TP53*), selected on the basis of their expression profile in RNA-seq/RT-qPCR and relevance in cell cycle regulation, was measured by RT-qPCR (Fig. 4C). Overall, the data showed that the expression profiles of the selected genes were similar between cells infected with AAV2 or UV-AAV2, respectively, but different from cells infected with scAAVeGFP. This observation is in accordance with previous studies, showing that infection with scAAV2 vectors allowed the cells to progress through mitosis, an event that occurred significantly less frequently upon infection with a single-stranded recombinant AAV2 vector (rAAV2) (28). Next, the capacity of AAV2 or rAAVeGFP, respectively, to induce a cell cycle arrest in NHF cells was confirmed by flow cytometry using propidium iodide (PI) staining (Fig. 4D and F) and confocal laser scanning microscopy (CLSM) using 4′,6-diamidino-2-phenylindole (DAPI) staining (Fig. 4E and G). The DAPI-based cell cycle analysis workflow was adapted from the protocol published by Roukos et al. (29,29) and validated as described previously (30). Both assays revealed an increase in the number of cells in the G2 cell cycle phase upon AAV2 (Fig. 4D and E) or rAAVeGFP (Fig. 4F and G) infection, indicating a G2 arrest. The shift from S to G2 cell cycle phase upon AAV2 or rAAVeGFP infection was even more pronounced at 48 hpi (see Fig. S3).

Overall, the *in vitro* analysis of the transcriptome data using different techniques correlated well and underscored the quality of the generated data.

### Upregulated genes in GO term innate immune response

**Fig 5:**
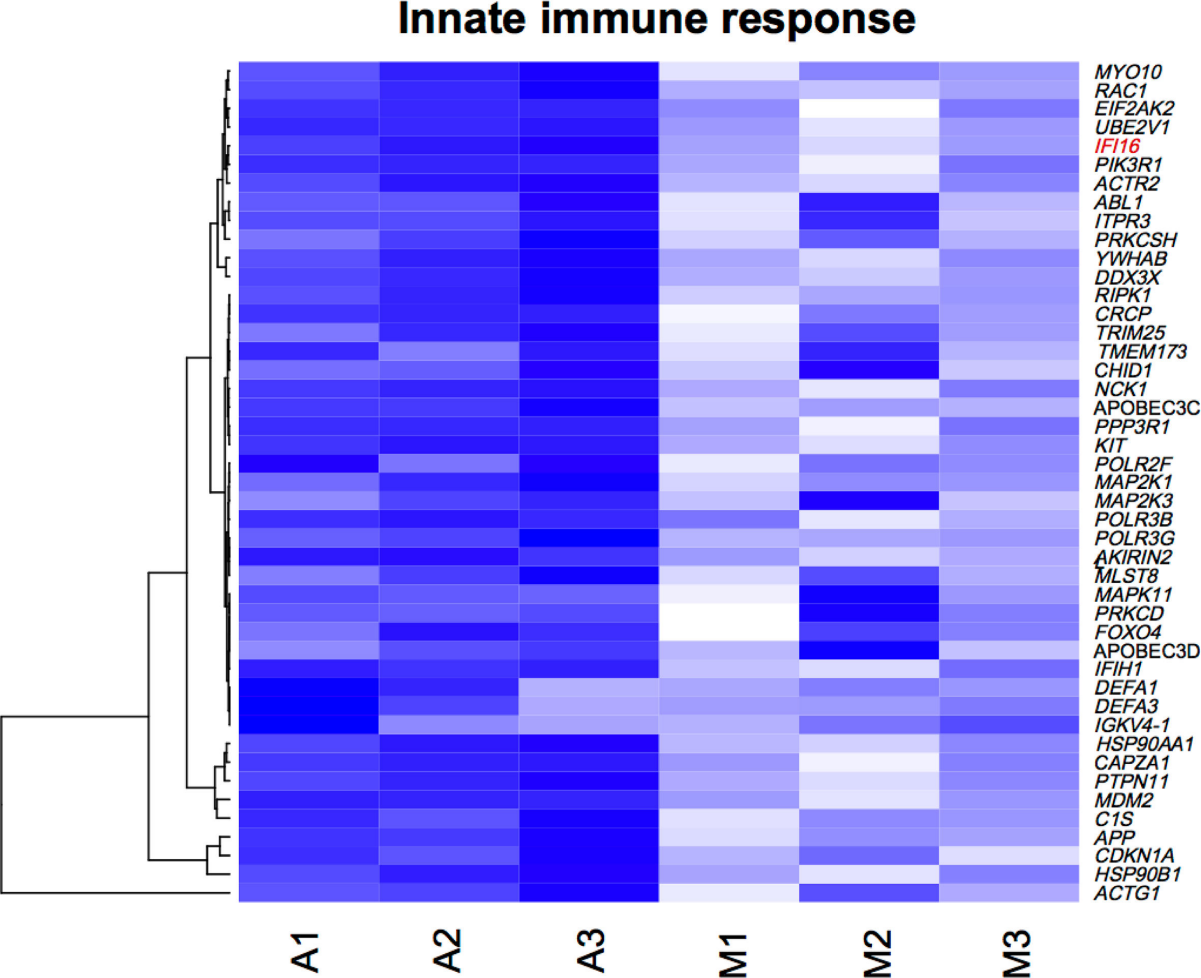
Heat map of upregulated genes in GO term innate immune response. Reads of the most differentially expressed genes (specified on the right of the heat map) between AAV2-infected (A1 to A3) and mock-infected NHF cells (M1 to M3). The dendrogram (left side of the heat map) illustrates the unsupervised clustering of the genes. The selected GOI (*IFI16*) is highlighted in red.

After having confirmed the quality and robustness of the transcriptome data, we focused on the GO term innate immune response (Fig. 5), which included, among others, the interferon-inducible p200-family protein IFI16, which is assumed to be an innate immune sensor for cytosolic and nuclear dsDNA as well as ssDNA (13). Besides, it has been shown that IFI16 can be a restriction factor for many different viruses through various mechanisms, including interferon response, transcriptional regulation, and epigenetic modifications (13–19). Hence, as the AAV2 DNA can be present as ssDNA, dsDNA, and circular dsDNA, it might be reasonable to expect that AAV2 infection may provoke an IFI16-triggered reaction.

### The post-transcriptional silencing of *IFI16* increases AAV2 transduction efficiency independent of the structure of the vector genome

**Fig 6:**
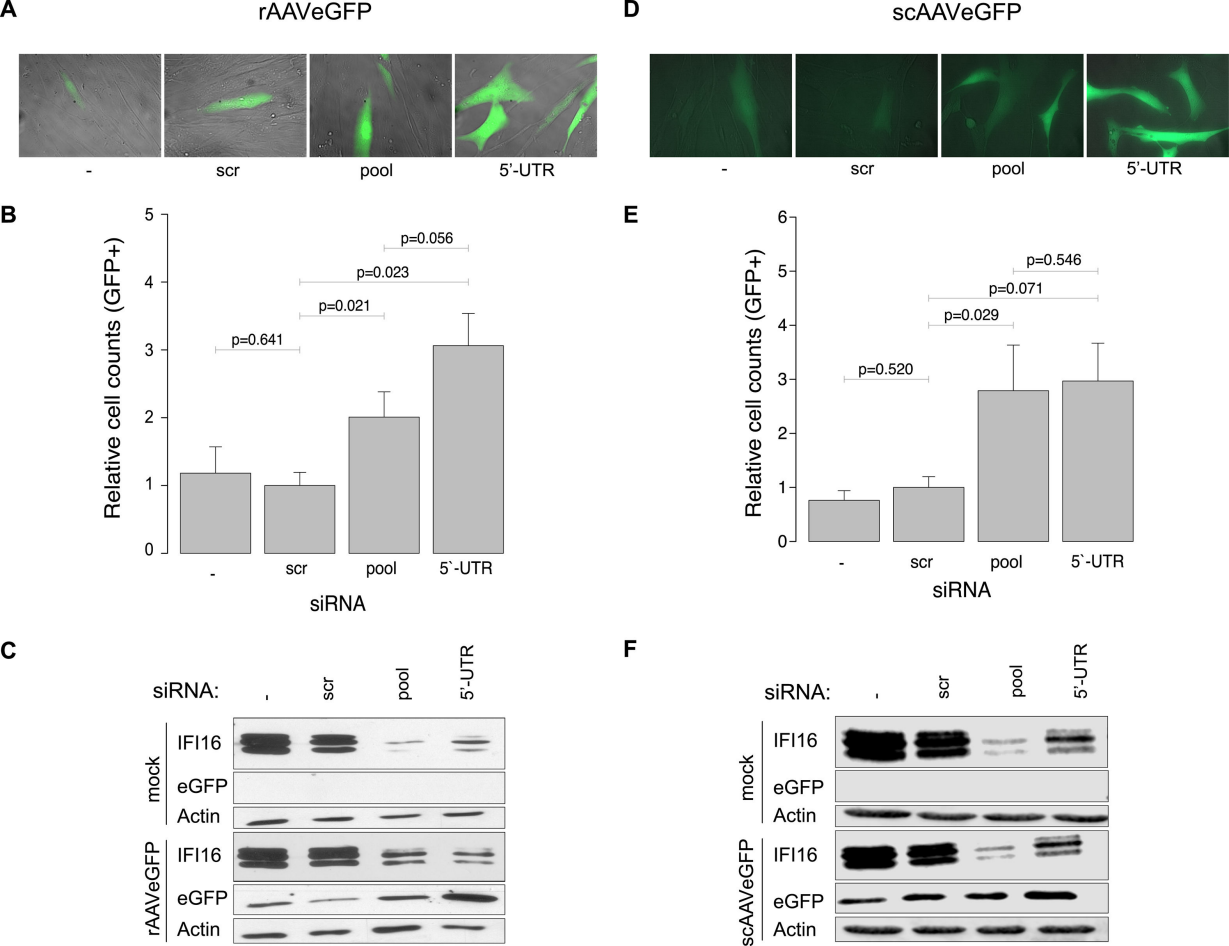
Post-transcriptional silencing of *IFI16* increases AAV2 transduction efficiency independent of the vector genome structure. NHF cells were transfected with no, scr control, or *IFI16* targeting siRNAs. At 40 hpt, cells were either mock-infected or infected with rAAVeGFP (MOI 4,000) or scAAVeGFP (MOI 2,000). (**A and D**) At 24 hpi, cells were counted using a fluorescence microscope. (**B and E**) Graphs show mean and SD of the relative cell count of GFP-positive NHF cells from triplicate experiments. *P*-values were calculated using an unpaired Student’s *t*-test (*P ≤ 0.05, **P ≤ 0.01, ***P ≤ 0.001, and ****P ≤ 0.0001). (**C and F**) Knock-down of *IFI16* was confirmed on protein level.

To assess whether IFI16 influences AAV2 vector-mediated cell transduction, NHF cells were transfected with no, scrambled (scr) control, or different siRNAs targeting *IFI16*, including a pool of three different siRNAs targeting the coding sequence (cds) of *IFI16* as well as a single siRNA targeting the 5′ untranslated region (5′-UTR) of *IFI16*. At 40 hours post-transfection (hpt), cells were either mock-infected or infected with rAAVeGFP or scAAVeGFP, and at 24 hpi, transduced cells were counted using a fluorescence microscope (Fig. 6A, B, D, and E). Total cell lysates prepared at 24 hpi were subjected to Western blot analysis in order to assess the eGFP (hereinafter referred to as GFP) protein level and to confirm the knock-down of *IFI16* (Fig. 6C and F). C911 siRNA controls were used to confirm that the observed increase of AAV transduction efficiency upon siRNA-mediated knock-down of *IFI16* was not due to off-target effects (see Fig. S4). One of the C911 siRNA controls, IFI16.3_C911, indeed appeared to knock-down IFI16, at least on the protein level (see Fig. S4C), suggesting an off-target effect of its corresponding counterpart (IFI16.3_cds) (31). Overall, the data implied an IFI16-mediated inhibition of AAV2 vector-mediated transduction, independent of the structure of the vector genome, i.e*.*, independent of whether a single-stranded or self-complementary vector genome configuration was chosen.

### The IFI16-mediated inhibition of AAV2 vector-mediated transduction is interferon signaling independent

**Fig 7:**
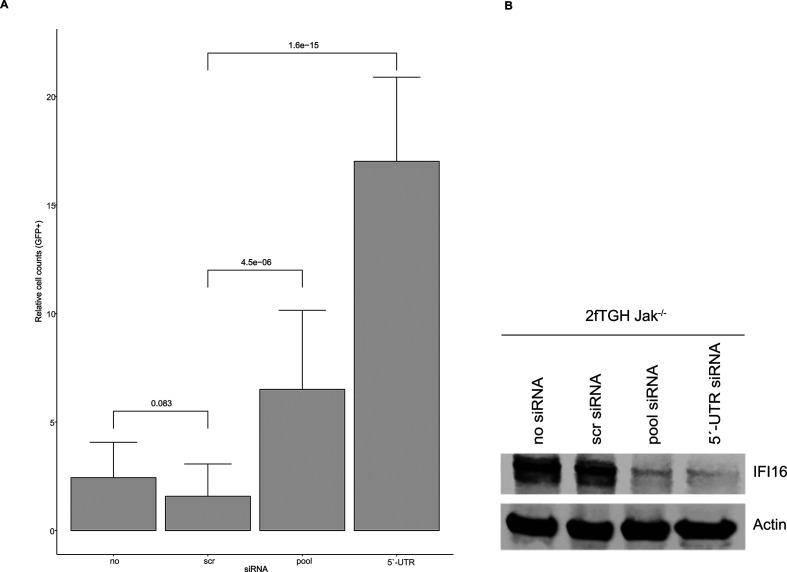
Role of interferon signaling. 2fTGH Jak1^-/-^ cells were transfected with no, scr control, or *IFI16* targeting siRNAs. At 40 hpt, cells were either mock-infected or infected with rAAVeGFP (MOI 1,000). At 24 hpi, transduced cells were counted by fluorescence microscopy. (**A**) Graph shows mean and SD of the relative cell count of GFP positive 2fTGH Jak1^-/-^ cells from triplicate experiments. *P*-values were calculated using an unpaired Student’s *t*-test (*P ≤ 0.05, **P ≤ 0.01, ***P ≤ 0.001, and ****P ≤ 0.0001). (**B**) Knock down of *IFI16* was confirmed on protein level.

To assess whether interferon signaling is relevant for the IFI16-mediated inhibition of AAV2 transduction, 2fTGH Jak1^-/-^ cells were reverse transfected with no siRNA, scr siRNA, a pool of three siRNAs targeting the coding sequence of *IFI16,* or a single siRNA targeting the 5′-UTR of *IFI16*. At 40 hpt, the cells were either mock-infected or infected with rAAVeGFP, and at 24 hpi, transduced cells were counted using a fluorescence microscope (Fig. 7A) and subsequently subjected to Western blot analysis to confirm the knock-down of *IFI16* (Fig. 7B). The results in Fig. 7A show an increase in rAAVeGFP transduction efficiency (pool, UTR) also in the Jak1^-/-^ cells, indicating an interferon signaling-independent mechanism of the IFI16-mediated inhibition of AAV2 vector-mediated transduction.

### The IFI16-mediated inhibition of AAV2 vector-mediated transduction is STING independent

**Fig 8:**
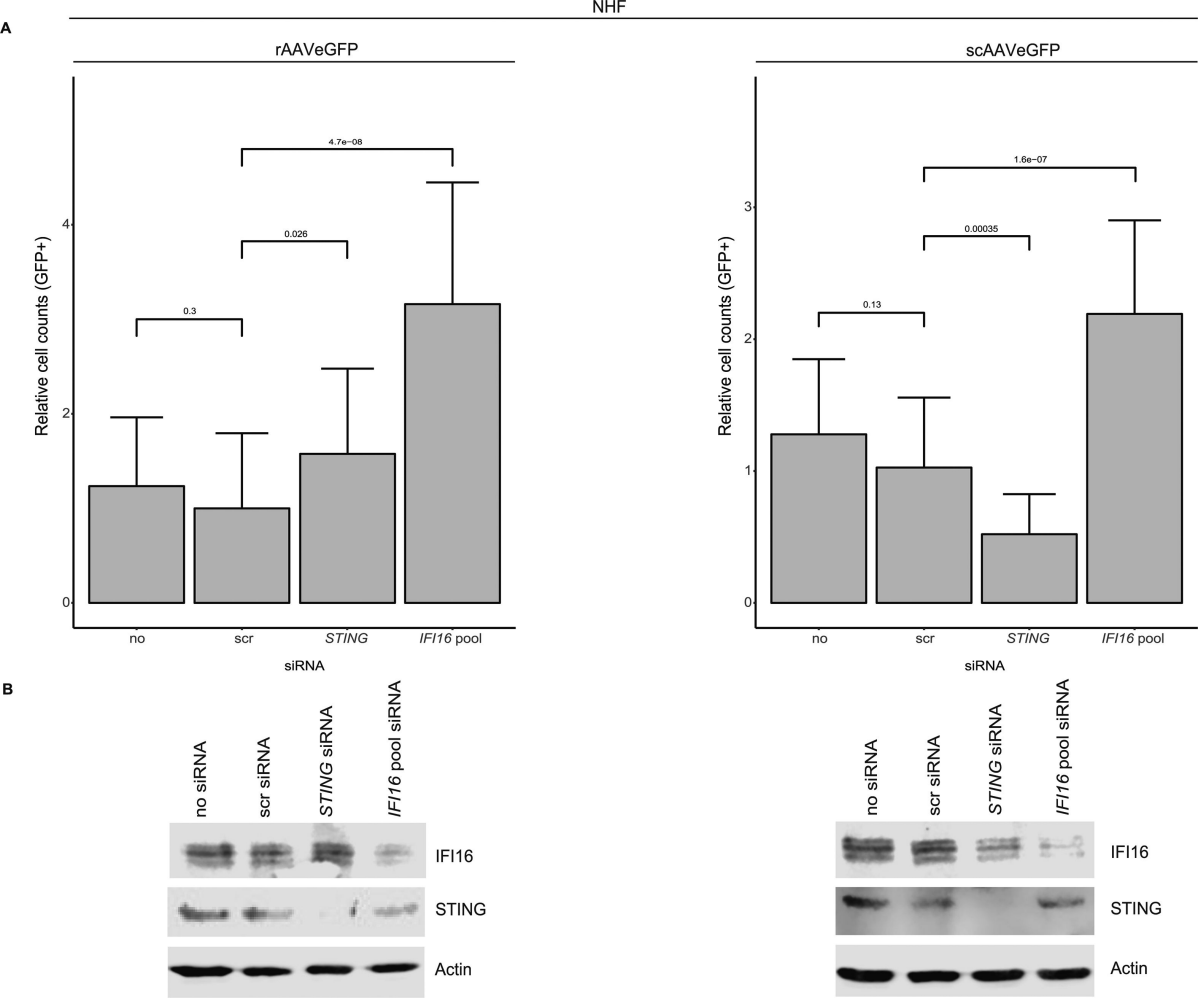
Effect of STING on AAV2 vector-mediated transduction. NHF cells were transfected with no, scr control, *STING,* or *IFI16* targeting siRNAs. At 40 hpt, cells were mock-infected or infected with either rAAVeGFP (MOI 6,000) or scAAVeGFP (MOI 4,000). At 24 hpi, transduced cells were counted by fluorescence microscopy. (**A**) Graphs show mean and SD of the relative cell count of GFP-positive NHF cells from triplicate experiments. *P*-values were calculated using an unpaired Student’s *t*-test (*P ≤ 0.05, **P ≤ 0.01, ***P ≤ 0.001, and ****P ≤ 0.0001). (**B**) Knock down of *IFI16* and *STING* was confirmed on protein level.

The cyclic GMP-AMP synthase (cGAS)-STING (stimulator of interferon genes) pathway plays a crucial role in a variety of viral infections, such as HSV-1, EBV, HPV, and HCV [reviewed in reference (32)]. The binding of cGAS to DNA provokes a conformational change of the active site, leading to the synthesis of 2ʹ3ʹ-cyclic GMP-AMP (2ʹ3ʹ-cGAMP). 2ʹ3ʹ-cGAMP operates as a second messenger that binds to the endoplasmic reticulum-membrane adaptor protein STING and induces a conformational change that results in the activation of STING, leading to the expression of type I interferon and pro-inflammatory cytokines in an IRF-3- or NF-κB-dependent manner. Upon induction, IFI16 translocates from the nucleus to the cytoplasm, where it interacts and activates STING [reviewed in reference (33)]. To address the question of functional STING signaling, different cell lines (NHF, U2OS, and HeLa) were treated with 2′3′-cGAMP (3 µM) for 9 h, and total RNA was extracted, converted to cDNA, and subjected to RT-qPCR using specific primers for *STING* and *ISG56* (see Fig. S5). Overall, the results showed no impairment of the STING pathway in NHF cells, whereas in U2OS and HeLa cells a deficient STING pathway was observed.To explore the role of STING in the IFI16-mediated inhibition of AAV vector transduction, NHF cells were transfected with no, scr, or different siRNAs, including a pool of three different siRNAs targeting *IFI16*, as well as single siRNA targeting the cds of *STING*. At 40 hpt, the cells were either mock-infected or infected with rAAVeGFP or scAAVeGFP, and at 24 hpi, the transduced cells were counted by using a fluorescence microscope (Fig. 8A) and subsequently subjected to Western blot analysis to confirm the knock down of *IFI16* and *STING* (Fig. 8B). Generally, the data indicated a STING-independent IFI16-mediated inhibition of AAV2 vector-mediated transduction.

### Exogenous complementation of *IFI16* in U2OS *IFI16*^-/-^ cells

**Fig 9:**
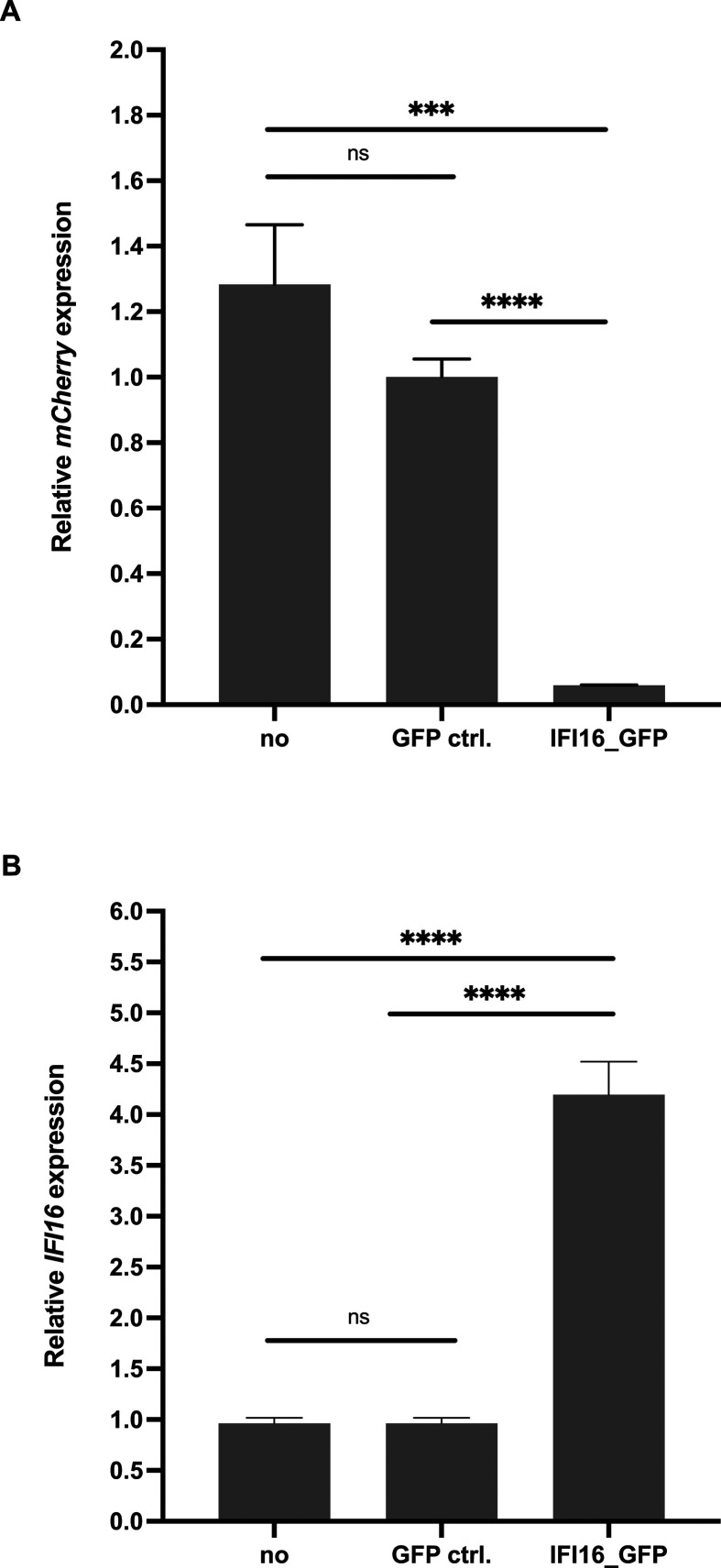
Exogenous complementation of *IFI16* in U2OS *IFI16*^-/-^ cells. U2OS IFI16^-/-^ were either untransduced, transduced with lentiviral vectors expressing GFP (MOI 5), or transduced with lentiviral vectors expressing *IFI16* fused to monomeric GFP (MOI 5) in the presence of polybrene. After 72 hours, the cells were infected with rAAV2mCherry (MOI 500). (**A**) mCherry expression was assessed by RT-qPCR using specific primers for mCherry. (**B**) Exogenous complementation of *IFI16* was confirmed on transcript level. Graphs show mean and SD of the relative gene expression from triplicate experiments. *P*-values were calculated using an unpaired Student’s *t*-test (*P ≤ 0.05, **P ≤ 0.01, ***P ≤ 0.001, and ****P ≤ 0.0001).

With respect to the impaired STING signaling in U2OS cells, an assay was established to exogenously complement *IFI16* in U2OS *IFI16*^-/-^ cells. To this end, U2OS IFI16^-/-^ cells were either untransduced (no), transduced with lentiviral vectors expressing GFP (GFP ctrl.), or transduced with lentiviral vectors expressing *IFI16* fused to monomeric GFP (IFI16_GFP). After 72 hours, the cells were infected with rAAVmCherry, and at 24 hpi, the cells were subjected to RT-qPCR (Fig. 9). Overall, the results showed a decrease in the relative mCherry expression (Fig. 9A) upon exogenous complementation of *IFI16* (Fig. 9B).

### Sub-nucleolar localization of IFI16

**Fig 10:**
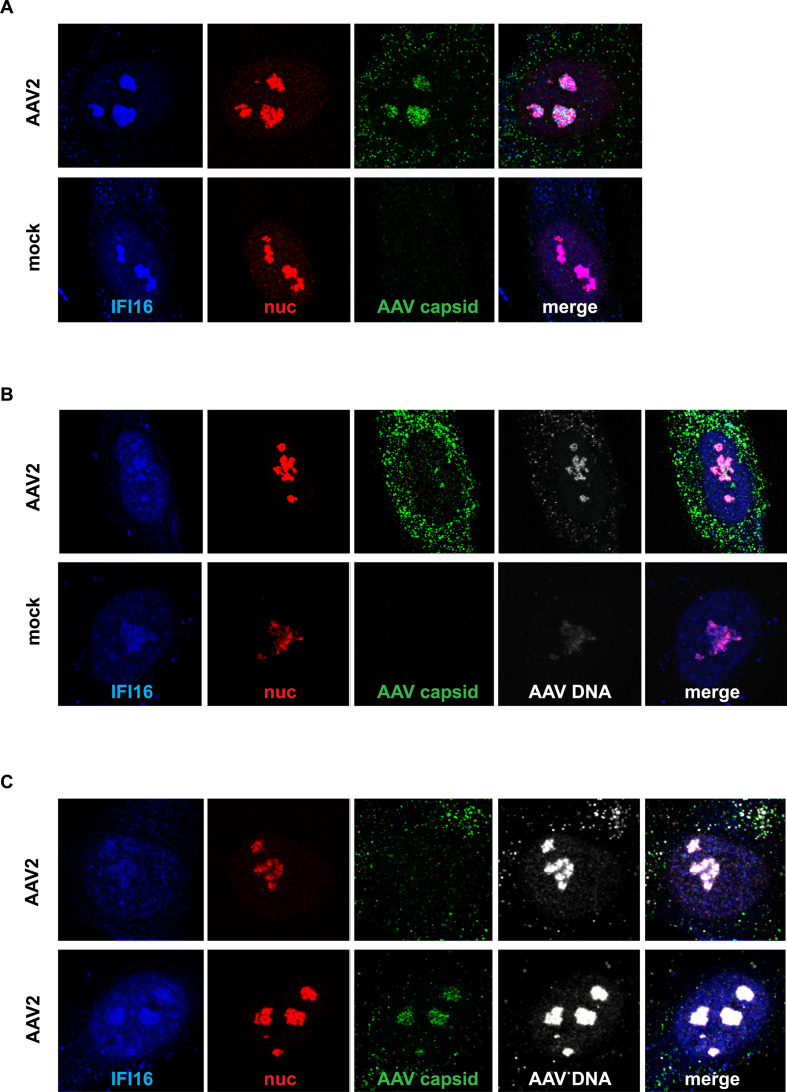
Multicolor IF combined with FISH. NHF cells were infected with AAV2 (MOI 20,000). After 24 h, the cells were fixed and processed for multicolor IF analysis combined with FISH and CLSM. IFI16 was detected by direct labeling of the antibody to ATTO-390 (blue). Nucleoli were visualized using an antibody against fibrillarin (red). Capsids were detected using an antibody against intact AAV2 capsids (green). AAV2 DNA (gray) was detected by linking the amine-modified DNA to AF647. (**A**) Nucleolar localization of IFI16 and AAV2 capsids. (**B**) Nucleolar localization of IFI16 and AAV2 DNA. (**C**) Nucleolar localization of IFI16, AAV2 DNA, and, conditionally, intact AAV2 capsids.

To assess the spatial distribution of IFI16 and AAV2 genomes, a combined immunofluorescence analysis (IF) and fluorescence *in situ* hybridization (FISH) was established. To this end, NHF cells were infected with AAV2, and 24 hpi, they were fixed and processed for multicolor IF analysis combined with FISH and CLSM. The results showed the accumulation of IFI16 in nucleoli, together with AAV2 DNA and, conditionally, AAV2 capsids (Fig. 10A through C; Fig. S6). However, the image-based quantification of the post-transcriptional silencing of *IFI16* revealed that partial uncoating in the cytoplasm, complete uncoating in the nucleolus, and cell cycle progression (30) were not affected (data not shown).

### The post-transcriptional silencing of *IFI16* increases AAV2 *rep* but not *cap* expression

**Fig 11:**
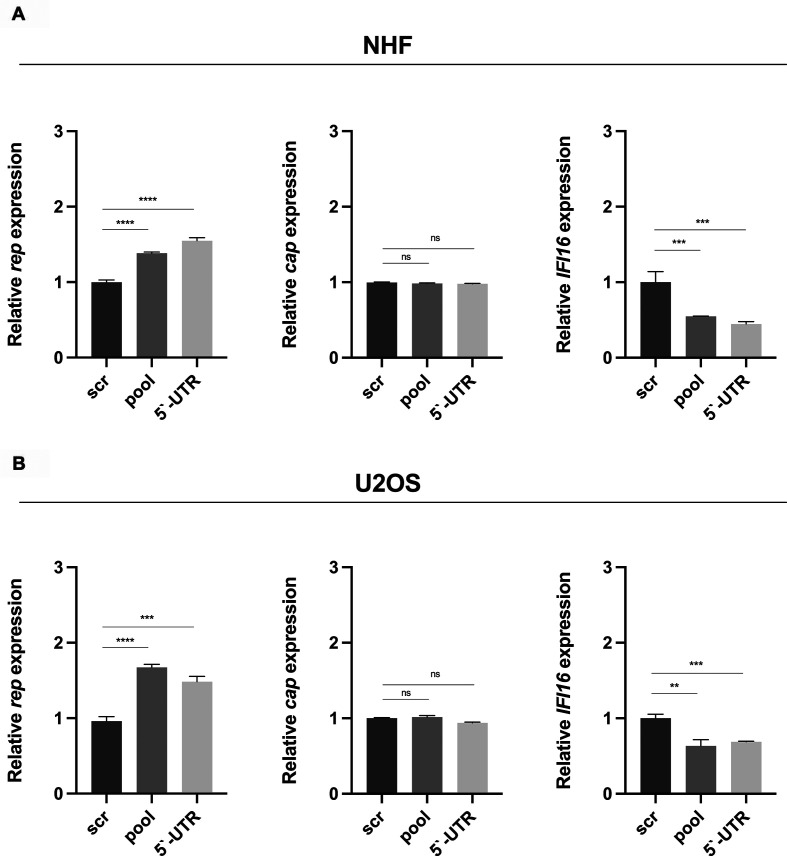
Post-transcriptional silencing of *IFI16* increases AAV2 *rep* but not *cap* expression. (**A**) NHF and (**B**) U2OS cells were transfected with scr control or *IFI16* targeting siRNAs, respectively. At 40 hpt, cells were infected with AAV2 (NHF; MOI 4,000, U2OS; MOI 2,000). At 24 hpi, total RNA was extracted and subjected to RT-qPCR using specific primers for the Rep helicase domain (*rep*), *cap* gene (*cap*), or *IFI16*. Graphs show mean and SD of the relative gene expression from triplicate experiments. *P*-values were calculated using an unpaired Student’s *t*-test (*P ≤ 0.05, **P ≤ 0.01, ***P ≤ 0.001, and ****P ≤ 0.0001).

**Fig 12:**
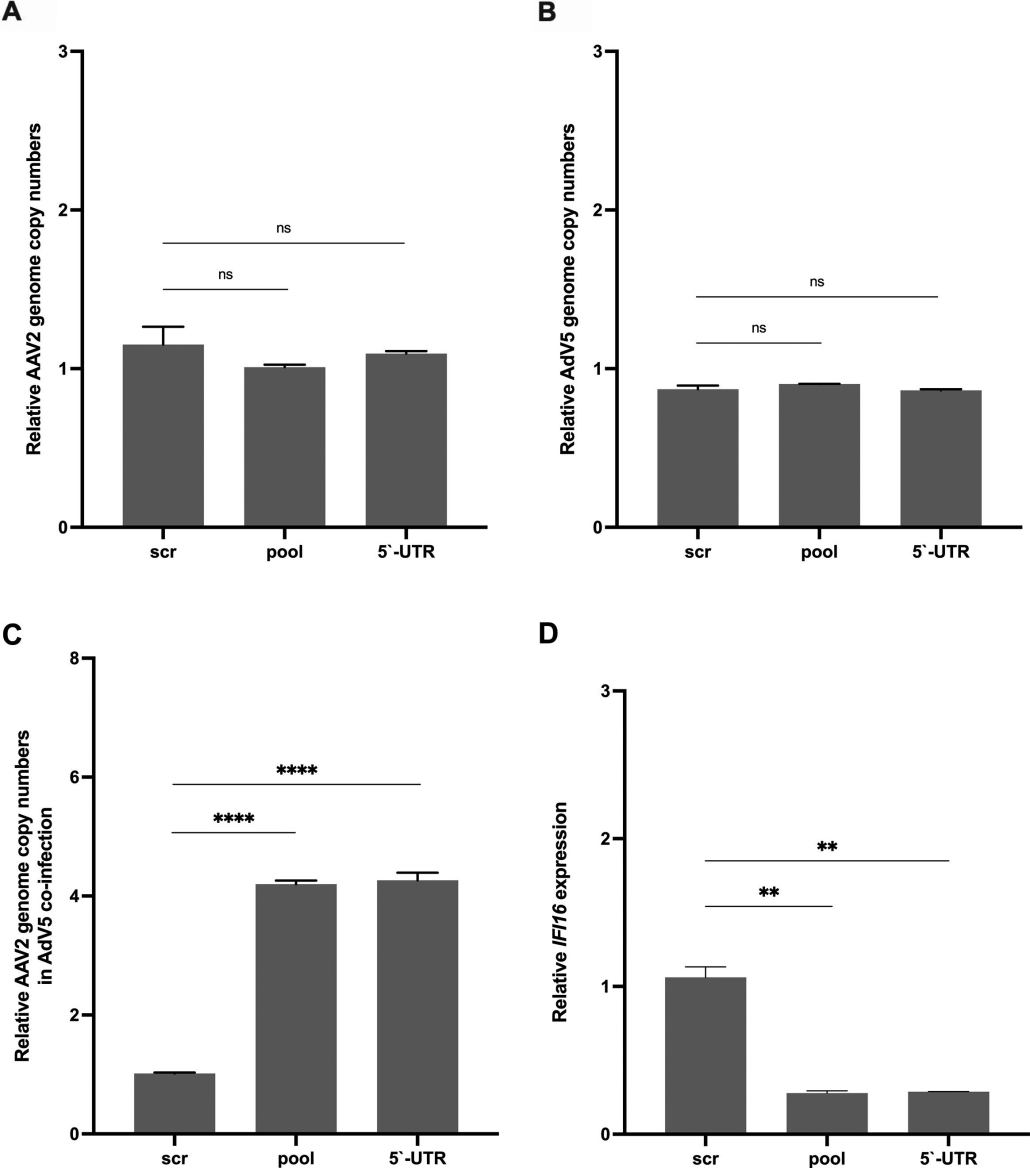
Post-transcriptional silencing of *IFI16* increases AAV2 genome replication in the presence of AdV5. NHF cells were transfected with scr control or *IFI16* targeting siRNAs, respectively. At 40 hpt, cells were either infected with (**A**) AAV2 (MOI 2,000), (**B**) AdV5 (MOI 5), or (**C**) co-infected with AAV2 (MOI 2,000) and AdV5 (MOI 5). After 24 h, total DNA was isolated and subjected to quantitative PCR using specific primers for AAV2 or AdV5, respectively. (**D**) Knock down of *IFI16* was confirmed on transcript level. Graphs show mean and SD of the relative genome copy numbers or the relative gene expression, respectively, from triplicate experiments. *P*-values were calculated using an unpaired Student’s *t*-test (*P ≤ 0.05, **P ≤ 0.01, ***P ≤ 0.001, and ****P ≤ 0.0001).

To explore the influence of IFI16 on the expression of AAV2-specific genes, *rep* and *cap*, NHF (Fig. 11A) and U2OS (Fig. 11B) cells were reverse transfected with either scr siRNA or different siRNAs, including a pool of three different siRNAs targeting cds of *IFI16* (pool), as well as single siRNA targeting the 5′-UTR of *IFI16*. At 40 hpt, cells were infected with AAV2, and 24 hours later, total RNA was extracted and subjected to RT-qPCR using specific primers for the Rep helicase domain (*rep*), *cap* gene (*cap*), or *IFI16*. In summary, the data showed an increase in *rep* but not *cap* expression upon knock down of *IFI16* in both NHF and U2OS cells, indicating that the IFI16-mediated effect on AAV2 gene expression varies intra-genomically. Besides, the post-transcriptional silencing of *IFI16* did not only result in an increase in *rep* expression but also enhanced AAV2 genome replication in the presence of adenovirus type 5 (AdV5; Fig. 12C) without affecting the relative genome copy numbers of AAV2 (Fig. 12A) or AdV5 (Fig. 12B).

### The post-transcriptional silencing of *IFI16* increases vector-mediated GFP expression

**Fig 13:**
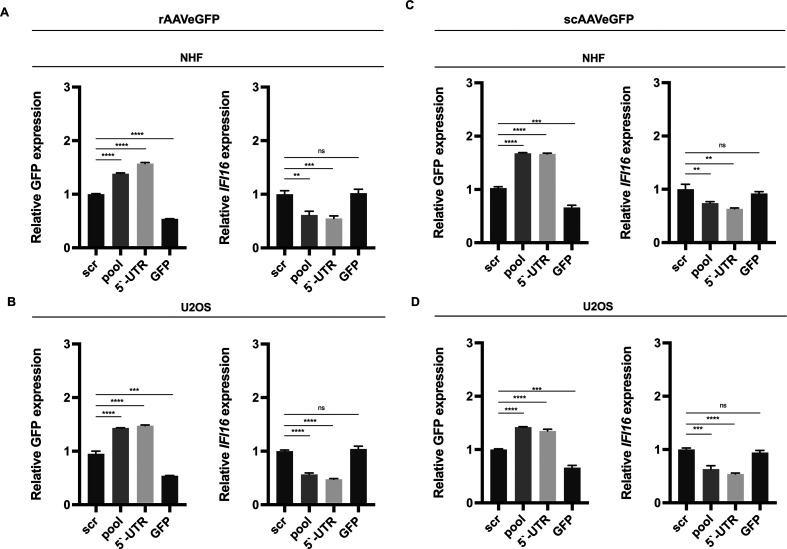
Post-transcriptional silencing of *IFI16* increases vector-mediated GFP expression. NHF and U2OS cells were transfected with scr control, GFP control, or *IFI16* targeting siRNAs. At 40 hpt, cells were infected either with (**A and B**) rAAVeGFP (NHF; MOI 4,000, U2OS; MOI 2,000) or (**C and D**) scAAVeGFP (NHF; MOI 2,000, U2OS; MOI 1,000). At 24 hpi, total RNA was extracted and subjected to RT-qPCR using specific primers for GFP or *IFI16*. Graphs show mean and SD of the relative gene expression from triplicate experiments. *P*-values were calculated using an unpaired Student’s *t*-test (*P ≤ 0.05, **P ≤ 0.01, ***P ≤ 0.001, and ****P ≤ 0.0001).

To assess the influence of IFI16 on vector-mediated GFP expression, NHF and U2OS cells were reverse transfected with either scr siRNA or different siRNAs, including a pool of three different siRNAs targeting the cds of *IFI16* (pool), as well as a single siRNA targeting the 5′-UTR of *IFI16* or a control siRNA targeting GFP. At 40 hpt, cells were either infected with rAAVeGFP (Fig. 13A and B) or scAAVeGFP (Fig. 13C and D), and 24 hours later, total RNA was extracted and subjected to RT-qPCR using specific primers for GFP or *IFI16*. In summary, the data showed a strong increase in GFP expression upon knock down of *IFI16* in both cell lines regardless of the structure of the vector genome configuration, single-stranded or self-complementary. Intriguingly, these results indicate that IFI16 affects not only wild-type gene expression (in a promoter-dependent manner) but also vector-mediated gene expression, suggesting an overarching IFI16-mediated gene regulation mechanism.

### IFI16 inhibits AAV2 gene expression in an Sp1-dependent manner

Emerging evidence implies that IFI16 exerts its effects via various genome regulation mechanisms, independently of innate immune sensing. For example, IFI16 promotes the addition of heterochromatin marks and yet reduces the number of euchromatin marks on specific viral genomes (15, 16), or it affects viral gene expression by reducing the availability of the transcription factor Sp1 (19).

**Fig 14:**
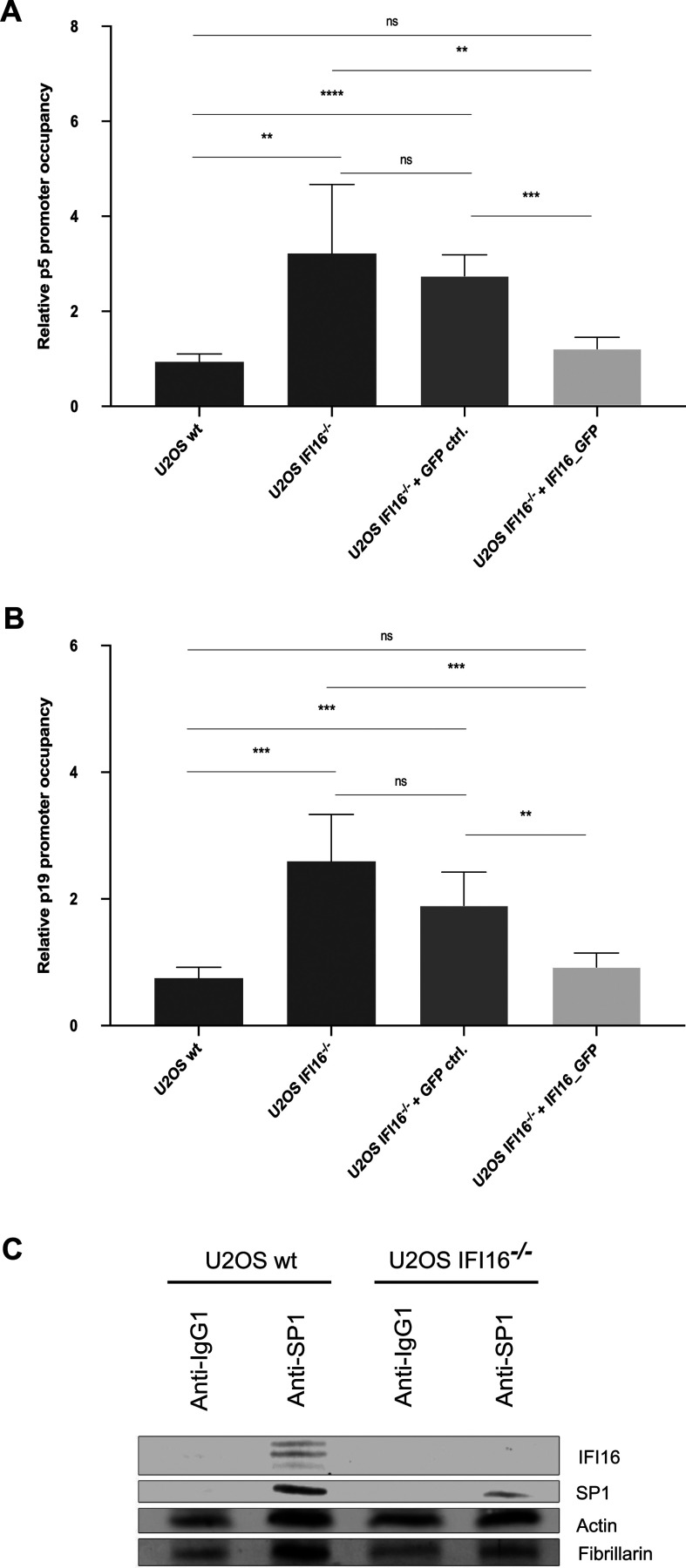
IFI16 inhibits AAV2 gene expression in an Sp1-dependent manner. U2OS IFI16^-/-^ and U2OS wt cells were either untransduced (U2OS IFI16^-/-^ or U2OS wt, respectively), transduced with lentiviral vectors expressing GFP (MOI 5; U2OS IFI16^-/-^ + GFP ctrl.), or transduced with lentiviral vectors expressing *IFI16* fused to monomeric GFP (MOI 5; U2OS IFI16^-/-^ + IFI16_GFP). After 72 hours, the cells were infected with AAV2 (MOI 20,000) and 24h later subjected to ChIP assays using an anti-Sp1 antibody and primers for the (**A**) p5 or (**B**) p19 promoter regions, respectively. (**C**) Interaction of IFI16 and Sp1 was assessed by co-immunoprecipitation in U2OS wt and U2OS IFI16^-/-^ cells. Graphs show mean and SD of the relative promoter occupancy from triplicate experiments. *P*-values were calculated using an unpaired Student’s *t*-test (*P ≤ 0.05, **P ≤ 0.01, ***P ≤ 0.001, and ****P ≤ 0.0001).

**Fig 15:**
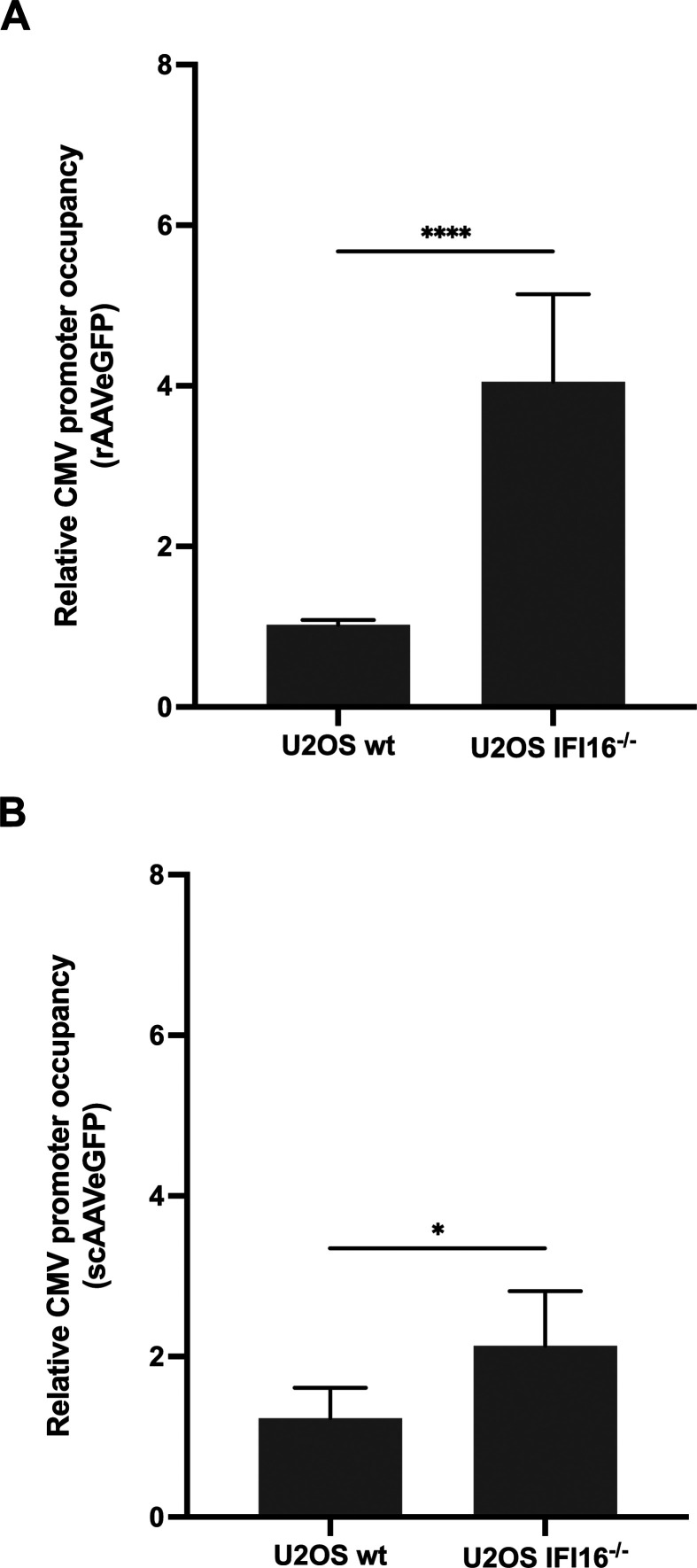
IFI16 inhibits AAV2 vector-mediated gene expression in an Sp1-dependent manner. U2OS IFI16^-/-^ cells or the parental cell line U2OS wt were infected with (**A**) rAAVeGFP (MOI 20,000) or (**B**) scAAVeGFP (MOI 20,000), and at 24 hpi, the cells were subjected to ChIP assays using an anti-Sp1 antibody and primers for CMV promoter regions. Graph shows mean and SD of the relative promoter occupancy from triplicate experiments. *P*-values were calculated using an unpaired Student’s *t*-test (*P ≤ 0.05, **P ≤ 0.01, *** *P* ≤ 0.001, and ****P ≤ 0.0001).

As both wild-type AAV2 and AAV2 vector genome expression was affected by the post-transcriptional silencing of *IFI16,* and as we did not observe any changes in methylation marks on either the wild-type or the vector genome (data not shown), we assessed whether IFI16 exerts its effect in a Sp1-dependent manner. To this end, U2OS IFI16^-/-^ or U2OS wild type (wt) cells were either untransduced (U2OS IFI16^-/-^ or U2OS wt, respectively), transduced with lentiviral vectors expressing GFP (U2OS IFI16^-/-^ + GFP ctrl.), or transduced with lentiviral vectors expressing *IFI16* fused to monomeric GFP (U2OS IFI16^-/-^ + IFI16_GFP) in the presence of polybrene. After 72 hours, the cells were either mock-infected or infected with AAV2 at a multiplicity of infection (MOI) of 20,000 and 24 h later subjected to chromatin immunoprecipitation (ChIP) assays using an anti-Sp1 antibody and primers for the p5 (Fig. 14A) or p19 (Fig. 14B) promoter regions, respectively. Intriguingly, the relative Sp1 promoter occupancy of p5 and p19 was significantly higher in the untransduced (U2OS IFI16^-/-^) or control vector-transduced cells (U2OS IFI16^-/-^ + GFP ctrl.) compared to the *IFI16* complemented cells (U2OS IFI16^-/-^ + IFI16_GFP) or the parental cell line (U2OS wt), respectively, indicating that IFI16 indeed restricts AAV2 independently of immune sensing by binding (Fig. 14C) and inhibiting the host transcription factor Sp1 that transactivates the viral promoter regions, thereby driving AAV2 gene expression. Moreover, the IFI16-mediated unavailability of Sp1 did not only affect the AAV2 rep promoters but also the CMV promoter in AAV2 vector genomes (Fig. 15). However, the relative CMV promoter occupancy of the self-complementary AAV2 vector (Fig. 15B) was less pronounced compared to the single-stranded vector (Fig. 15A), which might be reasoned by the nature of this promoter (minimal CMV), possessing less Sp1 binding sites than the full length CMV promoter of the single-stranded AAV2 vector. Overall, these data imply that IFI16 inhibits gene expression of wild-type AAV2 and AAV2 vectors by reducing the availability of the transcription factor Sp1, thereby reducing the SP1-mediated transactivation of the viral promoters.

## DISCUSSION

Downstream analysis of the RNA-seq data that included 1,930 genes with a *P*-value < 0.01 and more than 40 reads showed eight distinct clusters of biological processes, which differ between AAV2- and mock-infected NHF cells. The most prominent biological processes include the regulation of macromolecules/metabolic processes/gene expression and cell cycle regulation. Further analysis of the clusters with the aid of heat maps resulted in the identification of the 50 most differentially expressed genes. Of special note was the heat map for the chromatin organization, as it showed a maximum difference in reads (log2) of roughly |2|, representing a fold difference of 4 between AAV2- and mock-infected cells. A possible explanation for the downregulation of many histones could be that the histone deacetylase-2 (*HDAC2*) and the NAD-dependent deacetylase sirtuin-1 (*SIRT1*) genes are upregulated. Histone deacetylases remove acetyl groups, which results in an increase of positively charged histone tails. This leads to a high-affinity binding between the histones and DNA backbone, entailing a condensed DNA structure that prevents transcription. Further evidence was given by several upregulated histone methyltransferases, which transfer methyl groups from S-adenosyl methionine either on arginine or lysine residues of the H3 and H4 histones. Methylated histones can then either be transcriptionally active or repressed. It would be interesting to further evaluate the data in order to draw a conclusion whether the found methyltransferases act as silencers or activators and whether or not these aforesaid AAV2-induced histone modification profiles are due to the AAV2-mediated cell cycle arrest.

When excluding the genes with a fold change of < |1.5|, the list with the 1,930 DE genes was reduced to 872 DE genes, of which 268 were downregulated and 604 upregulated. To gain a first impression in terms of biological relevance, the 872 genes were projected onto KEGG pathways. The KEGG analysis revealed that around 80% of the genes of the surveillance system of the cell cycle are negatively regulated upon AAV2 infection. To further evaluate the 872 differentially expressed genes, 10 genes were selected due to their differential expression profile in AAV2-infected and mock-infected cells and based on their relevance in the before-mentioned downstream analysis. Both transcription and protein levels of the selected genes assessed by RT-qPCR and Western blot analysis, respectively, correlated well with the RNA-seq and connoted a differential gene regulation upon AAV2 infection. Moreover, the expression profiles of wild-type AAV2 and UV-inactivated AAV2-infected cells indicated a shift in cell cycle progression upon infection, while that of cells infected with a recombinant AAV2 with a self-complementary genome configuration (scAAVeGFP) did not. This finding is in accordance with previous observations (28), showing that infection with self-complementary AAV2 vectors allowed the cells to progress through mitosis, an event that occurred significantly less frequently upon infection with single-stranded rAAV2 vectors. However, recombinant single-stranded AAV2 vectors, similar to wild-type AAV2 (34), may induce cell cycle arrest more efficiently compared to self-complementary AAV2 vectors due to the single-stranded nature of the genome.

Further examination of the differentially expressed genes in the different GO terms revealed an upregulation of several genes in the GOterm innate immune response, including the interferon-inducible p200-family protein IFI16, which is assumed to be an innate immune sensor for cytosolic and nuclear dsDNA as well as ssDNA (13). IFI16 has been shown to be a restriction factor of many different viruses through several mechanisms, including epigenetic modifications and interferon response. For example, it was noted that IFI16 acts as a cytosolic immune sensor of HIV-1 DNA species in macrophages (35) and promotes interferon induction via the cyclic GMP-AMP synthase and stimulator of interferon genes pathway (36). Another study reported the IFI16-mediated sensing of HIV-1 reverse transcription intermediates, resulting in a caspase-1-dependent pyroptotic cell death of HIV-infected CD4+ T cells (37). Although IFI16 is thought to act as a cytosolic sensor of viral DNA, it has mainly been detected in the nucleus and was shown to interact with nuclear herpes viral DNA (16, 38, 39). The pyrin and HIN domain (PYHIN) containing proteins, such as IFI16, are also known to act as transcriptional regulators. Recent data showed that IFI16 inhibits HCMV transcription (14) and restricts HSV-1 replication by repressing viral gene expression independently of innate immune sensing (15, 40), thereby constituting an innate immune sensor for cytosolic and nuclear dsDNA as well as ssDNA. AAV2 DNA being present as ssDNA, dsDNA, and circular dsDNA might therefore provoke an IFI16-triggered reaction. Indeed, the post-transcriptional silencing of *IFI16* increased AAV2 transduction efficiency, regardless of the structure of the vector genome, indicating an IFI16-mediated inhibition of AAV2 vector transduction. This IFI16-mediated inhibition, however, was shown to be Jak1 and STING independent, suggesting an immune-modulatory independent mode of action. Our data, however, indicate a putative interplay of IFI16 and AAV2 gene expression, as the post-transcriptional silencing of *IFI16* increased AAV2 *rep* expression. Several studies showed that IFI16 exerts its effects by various genome regulation mechanisms, independently of innate immune sensing, including the change of methylation marks (15, 16) or by affecting viral gene expression by reducing the availability of the transcription factor Sp1 (19). Although it has long been known that Sp1 plays a key role in the Rep-mediated induction of wild-type AAV promoter regions (41, 42), its relevance in AAV2 biology has hardly been explored because it is commonly accepted that Sp1 is ubiquitously and constitutively expressed. However, our data indicate that IFI16 reduces the availability of Sp1, thereby suppressing the Sp1-mediated activity.

Overall, we present here the first transcriptome analysis of AAV2-infected human primary fibroblasts. The high quality of the raw data allowed a broad and comparative analysis of the transcription profiles of mock-infected and AAV2-infected cells in the absence of a helper virus. Most importantly, our findings provide evidence that not only Toll-like receptor 9 detects AAV genomes and triggers an antiviral state upon AAV infection (43, 44), along with the transgenic genome-derived dsRNA-induced MDA5-mediated innate immune response (45), but also additional sensors such as IFI16 constitute other lines of antiviral defense by suppressing viral gene expression in an Sp1-dependent manner. Hence, further studies on the role of other PYHIN proteins as effectors of antiviral defense mechanisms in AAV2 infection or AAV vector-mediated cell transduction seem highly warranted.

## MATERIALS AND METHODS

### Cells

Normal human fibroblastcells (kindly provided by X. O. Breakefield, Massachusetts General Hospital, Charlestown, MA, USA) and HeLa cells were maintained in growth medium containing Dulbecco’s modified Eagle medium (DMEM) supplemented with 10% fetal bovine serum (FBS), 100 U/mL penicillin G, 100 µg/mL streptomycin, and 0.25 µg/mL amphotericin B (1% AB) at 37°C in a 95% air-5% CO_2_ atmosphere. IFI16 knock-out human bone osteosarcoma epithelial cells (U2OS IFI16^-/-^), as well as the parental cell line (U2OS wt), were kindly provided by Dr. Bala Chandran (Chicago Medical School, RFUMS, USA) and cultured in growth medium containing DMEM supplemented with GlutaMax, 10% FBS, and 1% AB at 37°C in a 95% air-5% CO_2_ atmosphere. 2fTGH Jak1^-/-^ cells (UA4 cell line, 12021505, Sigma-Aldrich, Merck KGaA, Darmstadt, Germany) were maintained in growth medium containing DMEM supplemented with GlutaMax, 10% FBS, and 1% AB at 37°C in a 95% air-5% CO_2_ atmosphere.

### Viruses

Wild-type AAV2 was produced by H. Büning (Hannover Medical School, Hannover, Germany). UV-irradiated AAV2 (UV-AAV2) was produced by UV inactivation of wtAAV2 with 254 nm UV light at a dose of 960 mJ/cm^2^ carried out in a UVC 500 UV cross-linker (Hoefer, Inc., San Francisco, CA, USA). UV inactivation was assessed on protein level using an anti-Rep antibody (data not shown). Recombinant (r)AAVeGFP, rAAVmCherry, and self-complementary (sc)AAVeGFP vectors of AAV serotype 2 were produced by transient transfection of 293T cells with pDG (46) and pAAVeGFP (kindly provided by M. Linden, King’s College London School of Medicine, London, UK), pAAVmCherry or pscAAVeGFP (kindly provided by J. Neidhardt, University of Zurich, Switzerland), respectively, and purified by an iodixanol density gradient. Titers of genome-containing particles were determined by quantitative PCR (qPCR) (47). Lentiviral vectors expressing GFP (TR30021V) or *IFI16* fused to monomeric GFP (RC202193L2V) were obtained from OriGene (Rockville, USA).

### Virus infection for RNA sequencing

5 × 10^6^ NHF cells were seeded into 10-cm tissue culture plates. The following day, the cells were either mock-infected or infected with AAV2 at a multiplicity of infection (MOI) of 500 in DMEM (0% FBS, 1% AB; pre-cooled to 4°C). The virus was allowed to adsorb at 4°C for 30 min before cultures were placed for 1 h into a humidified incubator at 37°C in a 95% air-5% CO_2_ atmosphere. After washing the cells with phosphate-buffered saline (PBS) and adding fresh medium (DMEM supplemented with 2% FBS and 1% AB), the cells were placed back at 37°C in a 95% air-5% CO_2_ atmosphere.

### RNA extraction

Cells were infected as described above. After 24 h, the total RNA was extracted using the Direct-zol RNA MiniPrep Kit according to the instructions of the manufacturer (Zymo Research Corp, Irvine, CA, USA). DNA was digested by adding 8 µL of 10× DNase buffer, 5 µL of DNase, 3 µL of RNase-free water, and 64 µL of RNA wash buffer and incubated for 15 min at 37°C. The samples were then purified according to the manufacturer’s (Zymo Research Corp) protocol. The quality and quantity of the extracted RNA were assessed using Bioanalyzer 2100 (Agilent Technologies, Inc., Santa Clara, CA, USA). Samples with an RNA integrity number of at least 8.3 were further used for the RNA-seq analyses.

### Illumina RNA sequencing

The RNA-seq experiment was performed in four steps: (i) a cDNA library was prepared from the RNA, (ii) cDNA was amplified in clusters, (iii) clusters were sequenced, and (iv) primary sequencing data were analyzed.

### Library preparation

The Illumina TruSeq Stranded Total RNA Sample Prep Kit with Ribo-Zero Human/Mouse/Rat protocol (Illumina, Inc. San Diego, CA, USA) was used for the following steps: 1 µg of total RNA was freed of cytoplasmic rRNA using biotinylated, Human/Mouse/Rat-specific oligonucleotides combined with Ribo-Zero rRNA removal beads and further fragmented into small pieces by divalent cations under elevated temperatures. First-strand cDNA was synthesized using Reverse Transcriptase II, Actinomycin D, and random primers. Second-strand cDNA synthesis was achieved by removing the RNA template and synthesizing a replacement strand, which incorporates dUTP instead of dTTP to generate double-stranded cDNA. The resulting cDNA samples were fragmented, 3′ adenylated and ligated to multiple indexing adaptors. Fragments containing those adaptors on both ends were selectively enriched using PCR. The quality and quantity of the enriched libraries were validated using Bioanalyzer 2100 (Agilent Technologies). Diluted libraries (10 nM) were pooled and further used for cluster generation.

### Cluster generation and sequencing

The TruSeq SR Cluster Kit v3-cBot-HS (Illumina, Inc.) was used for cluster generation using diluted (10 nM) and pooled libraries. Sequencing was performed on the Illumina HiSeq 2500 in the high throughput mode. Library preparation and sequencing were performed at the Functional Genomics Center Zurich (FGCZ) core facility

### Sequencing data analysis

Reads were aligned with the STAR aligner (STAR: ultrafast universal RNA-seq aligner with the additional parameters -- outFilterMatchNmin 30 - outFilterMismatchNmax 5 -- outFilterMismatchNoverLmax 0.05 -- outFilterMultimapNmax 50), which means that at least 30 bp matching is required and that at most five mismatches and 5% of mismatches are accepted. Read alignments were only reported for reads with less than 50 valid alignments. The Human genome build and annotation from Ensembl (GRCh37) was used as a reference. Spliced junctions derived from the Ensemble gene annotations. Additionally, the reference was extended to contain the AAV2 sequence (GenBank accession no. NC_001401). Expression counts were computed using the R Bioconductor package GenomicRanges (48). Differential expression was computed using the R DESeq2 package (49).

### Bioinformatic analyses

Gene Ontology term biological process analysis was performed by DAVID (22). An enrichment map of the DAVID GO terms BP analysis was constructed using the Cytoscape module Enrichment Map. Heat maps of the genes representing selected ontologies were constructed using R KEGG pathway analysis (R Bioconductor package Pathview) (50).

### Antibodies

The following primary antibodies were used: anti-β-actin (Sigma-Aldrich A5316; dilution for Western blottingWB; 1:10,000), anti-cyclin A (BD Biosciences; dilution for WB; 1:250), anti-cyclin B1 (Cell Signaling 4138; dilution for WB; 1:1,000), anti-CDK1 (Abcam; dilution for WB; 1:1,000), anti-E2F1 (Cell Signaling 3742; dilution for WB; 1:1,000), anti-p53 (Abcam; dilution for WB; 1:1,000), anti-Rb (Cell Signaling 9309; dilution for WB; 1:2,000), anti-Rb-P-S807/811 (Cell Signaling 8516; dilution for WB; 1:1,000), anti-IFI16 (Santa Cruz Biotechnology 1G7; dilution for WB; 1:500, dilution for immunofluorescence; 1:250), anti-STING (Santa Cruz Biotechnology E-20; dilution for WB; 1:500), anti-Rep (RDI, Division of Fitzgerald Industries; dilution for WB; 1:200), anti-AAV2 intact particle (A20, ProGen: dilution for IF; 1:50), anti-fibrillarin (Abcam ab5821; dilution for WB; 1:650), anti-Sp1 [Abcam ab227383; ChIP and co-immunoprecipitation (Co-IP) assays; 1.5 µg, dilution for WB; 1:500], or rabbit IgG1 antibody (Abcam ab171870; ChIP and Co-IP assays; 1.5 µg). The following secondary antibodies were used: rabbit-anti mouse IgG-horseradish peroxidase (HRP; SouthernBiotech; dilution 1:10,000) and goat-antirabbit IgG-HRP (SouthernBiotech; dilution: 1:10,000)

### Western blotting

A total of 1.5 × 10^6^ NHF cells were seeded into 10-cm tissue culture plates. The following day, the cells were either mock-infected or infected with AAV2 (MOI 500) in DMEM (0% FBS and 1% AB; pre-cooled to 4°C). The virus was allowed to adsorb at 4°C for 30 min before cultures were placed for 1 h into a humidified incubator at 37°C in a 95% air-5% CO_2_ atmosphere. After washing the cells with PBS and adding fresh medium (DMEM supplemented with 2% FBS and 1% AB), the cells were placed back at 37°C in a 95% air-5% CO_2_ atmosphere. After 48 h, the cells were trypsinized, washed once with PBS, and centrifuged for 5 min at 2,000 × *g* and 4°C. The pellet was dissolved in 100 µL protein loading buffer (2.5% SDS, 5% β-mercaptoethanol, 10% glycerol, 0.002% bromophenol blue, and 62.5 mM Tris-HCl, pH 6.8) and then the samples were boiled for 10 min. Cell lysates were separated, depending on the molecular weight of the protein of interest, on 10% or 12% SDS-polyacrylamide gels and transferred to Protran nitrocellulose membranes (Whatman, Bottmingen, Switzerland). Membranes were blocked with PBS-T (PBS containing 0.3% Tween 20) supplemented with 5% nonfat dry milk for 1 h at room temperature (RT). Incubation with antibodies was carried out with PBS-T supplemented with 2.5% milk. Primary antibodies were incubated overnight at 4°C, while secondary antibodies were incubated for 1 h at RT. Membranes were washed three times with PBS-T for 10 min after each antibody incubation step. HRP-conjugated secondary antibodies were detected by incubation with ECL (WesternBright ECL-spray, Advansta Inc., Menlo Park, CA, USA) for 2 min. The membranes were exposed to chemiluminescence detection films (Roche Diagnostics, Rotkreuz, Switzerland). Detection of anti-actin served as a loading control for the lysate.

### Quantitative reverse transcription PCR

For primer design, the Primer-BLAST tool, the Harvard primerbank, and the PrimerCheck of the SpliceCenter were used. To test the primers, a standard RT-PCR (with or without RT reaction) with mock-infected NHF cells was performed. The cycling protocol started with a denaturation step of 3 min at 95°C, followed by 37 cycles of 30 s at 94°C, 30 s at 55°C, and 1 min at 72°C followed by a final step of 10 min at 72°C. Subsequently, the reactions were analyzed on 1% agarose gel. Bands were expected between 100 and 200 bp, depending on the primer pair. The concentration and purity of RNA were determined by Qubit fluorometer analysis. To generate cDNA, the extracted RNA was reverse transcribed using the reverse transcription system (Promega Corporation, Fitchburg, WI, USA). For this, the following components were mixed: 4 µL MgCl_2_, 2 µL 10× RT buffer, 2 µL dNTPs (10 mM), 0.5 µL of the RNase inhibitor RNAsin, 0.65 µL AMV RT, 1 µL random or Oligo(dT) primers, 1 µg RNA, and RNase-free H_2_O in a total volume of 20 µL. The mixture was incubated for 10 min at room temperature and 15 min at 42°C. For enzyme inactivation, the sample was incubated for 5 min at 95°C and then incubated on ice for 5 min. A volume of 4 µL of the cDNA (approximately 10 ng) was used for qPCR and the rest was stored at −20°C. For each reaction, the following mixture was prepared: 1 µL of forward primer (10 µM), 1 µL of reverse primer (10 µM), 10 µL of SYBR Green PCR master mix, and 4 µL ddH_2_O and transferred into a well of a Hard-Shell 96-well PCR plate (MicroAmp fast 96-well reaction plate). A volume of 4 µL of the appropriate cDNA was added, and the 96-well plate was centrifuged for 1 min at 1,000 × *g* and subsequently run at the standard 20 µL qPCR SYBR green program on QuantStudio 3 real-time system (Applied Biosystem, ThermoFisher Scientific, Waltham, MA, USA). The experiment was performed as technical triplicates for each primer pair for infected and non-infected samples. GAPDH and SDHA were used as housekeeping genes for further normalization of the RT-qPCR raw data. The primer sequences used are listed in Table 2.

**TABLE 2:** RT-qPCR primers used in this study

**Gene**	**Primers**
*CCNB1*	5′-TGGGTCGGCCTCTACCTTTG-3′ (forward)
	5′-TGTTGCTCGACATCAACCTCTC-3′ (reverse)
*CCNA2*	5′-GGATGGTAGTTTTGAGTCACCAC-3′ (forward)
	5′-CACGAGGATAGCTCTCATACTGT-3′ (reverse)
*TP53*	5′-TTCCGAGAGCTGAATGAGGC-3′ (forward)
	5′-CTTCAGGTGGCTGGAGTGAG-3′ (reverse)
*E2F1*	5′-CATCCCAGGAGGTCACTTCTG-3′ (forward)
	5′-GACAACAGCGGTTCTTGCTC-3′ (reverse)
*CDK1*	5′-AAGCCGGGATCTACCATACC-3′ (forward)
	5′-CATGGCTACCACTTGACCTG-3′ (reverse)
*RB1*	5′-CTTGCATGGCTCTCAGATTCAC-3′ (forward)
	5′-AGAGGACAAGCAGATTCAAGGTG-3′ (reverse)
*CDKN1A*	5′-CCTGTCACTGTCTTGTACCCT-3′ (forward)
	5′-GCGTTTGGAGTGGTAGAAATCT-3′ (reverse)
*IFI16*	5′-CCAGCACAACCTTCCCTGAGAGCCATCT-3′ (forward)
	5′-GAAACTGCTGCTTGGTGTTGATGGAGGC-3′ (reverse)
GFP	5′-CCGAGGTGAAGTTCGAGG-3′ (forward)
	5′-GCCGTTCTTCTGCTTGTC-3′ (reverse)
*STING*	5′-TTCGAACTTACAATCAGCATTACAA-3′ (forward) (51)
	5′-CTCATAGATGCTGTTGCTGTAAACC-3′ (reverse) (51)
*ISG56*	5′-GGAAAAAAAGCCCACATTTGAGGT-3′ (forward) (51)
	5′-CTTTTGAAATTCCTGAAACCGACCA-3′ (reverse) (51)
mCherry	5′-GAACGGCCACGAGTTCGAGA-3′ (forward)
	5′-CTTGGAGCCGTACATGAACTGAGG-3′ (reverse)
AAV2 Rep	5′-ATTGACGGGAACTCAACG-3′ (forward)
	5′-ATTCATGCTCCACCTCAA-3′ (reverse)
GFP	5′-AAGGGCATCGACTTCAAGG-3′ (forward)
	5′-TGCTTGTCGGCCATGATATAG-3′ (reverse)
AAV2 Cap	5′-TTGAGGACGTTCCTTTCC-3′ (forward)
	5′-TGAAGGTGGTCGAAGGATTC-3′ (reverse)
*GAPDH*	5′-TGCACCACCAACTGCTTAGC-3′ (forward) (52)
	5′-GGCATGGACTGTGGTCATGAG-3′ (reverse) (52)
*SDHA*	5′-TGGGAACAAGAGGGCATCTG-3′ (forward) (52)
	5′-CCACCACTGCATCAAATTCATG-3′ (reverse) (52)

### Quantitative PCR

For qPCR, total DNA was isolated by using the DNeasy blood and tissue kit (Qiagen, Hilden, Germany) according to the manufacturer’s protocol. A volume 4 µL of the isolated DNA (approximately 10 ng) was used for qPCR and the rest was stored at −20°C. For each reaction, the following mixture was prepared: 1 µL forward primer (10 µM), 1 µL reverse primer (10 µM), 10 µL of SYBR Green PCR master mix, and 4 µL ddH_2_O and transferred into a well of a Hard-Shell 96-well PCR plate (MicroAmp fast 96-well reaction plate). A volume of 4 µL of the appropriate DNA was added, and the 96-well plate was centrifuged for 1 min at 1,000 × *g* and subsequently run at the standard 20 µL qPCR SYBR green program on QuantStudio 3 real-time system (Applied Biosystem, ThermoFisher Scientific, Waltham, MA, USA). The experiment was performed as technical triplicates for each primer pair for infected and non-infected samples. The transcriptional start site (TSS) of GAPDH was used as endogenous control. The primer sequences used are listed in Table 3.

**TABLE 3:** qPCR primers used in this study

**Gene**	**Primers**
AdV5	5′-CTGTGATGCTGGATGTGACC′ (forward) (53)
	5′-TGCTTCCATCAAACGAGTTG-3′ (reverse) (53)
AAV2 Rep	5′-ATTGACGGAACTCAACGAC-3′ (forward)
	5′-CCTC AACCACGTCCTTT-3′ (reverse)
*GAPDH TSS*	5′-TTCGACAGTCAGCCGCATCTTCTT-3′ (forward) (15)
	5′-CAGGCGCCCAATACGACCAAATC-3′ (reverse) (15)

### Co-immunoprecipitation

1.3 × 10^6^ U2OS IFI16^-/-^ or U2OS wt were washed twice with cold PBS, harvested using a cell scraper, and transferred into a 15 mL conical tube while being kept on ice. Next, the cells were pelleted at 900 *g* for 10 min at 4°C. The remaining pellet was resuspended in 50 µL of PBS and 100 µL of 2× SDS lysis buffer [100 mM Tris-HCl, pH 8.1, 2% SDS (wt/vol), and 20 mM EDTA] with protease inhibitor (cOmplete mini, Roche Cat. 11836153001). After 15 min of incubation on ice, the samples were centrifuged again, and the lysate was diluted 1:10 in dilution buffer [16.7 mM Tris-HCl, pH 8.1, 167 mM NaCl, 0.01% SDS (wt/vol), 1.2 mM EDTA, 1.1% Triton X-100 (vol/vol)]. Next, 80 µL of agarose protein G with salmon sperm DNA slurry (Millipore Cat. 16-201) was added for pre-clearance, and the samples were incubated with gentle agitation at 4°C for 30 min. The slurry was then centrifuged for 3 min, 4°C at 300 *g* (to not break the agarose beads), and the supernatant was recovered and split into two equal fractions. The fractions were then incubated either with 1.5 µg of anti-Sp1 antibody or rabbit IgG1 antibody overnight at 4°C with gentle agitation. The next day, 120 µL of agarose protein G with salmon sperm DNA slurry was added and incubated for 30 min at 4°C. The centrifugation for the following washing steps was performed at 4°C at 300 *g* for 3 min each. First, the samples were washed twice with low salt wash buffer [20 mM Tris-HCl, pH 8.1, 150 mM NaCl, 0.1% SDS (wt/vol), 2 mM EDTA, and 1% Triton X-100 (vol/vol)] and then with high salt wash buffer [20 mM Tris-HCl, pH 8.1, 500 mM NaCl, 0.1% SDS (wt/vol), 2 mM EDTA, and 1% Triton X-100 (vol/vol)]. Next, the samples were washed with LiCl salt wash buffer [20 mM Tris-HCl, pH 8.1, 250 mM LiCl, 1% deoxycholate (wt/vol), 1 mM EDTA, and 1% Nonidet P-40 (vol/vol)], and finally twice with TE (pH 8.0). After centrifugation, the supernatant was discarded, and the samples were dissolved in 6× protein loading buffer, boiled for 10 min, and subjected to Western blot analysis.

### Chromatin immunoprecipitation

5 × 10^5^ U2OS IFI16^-/-^ or U2OS wt were either untransduced or transduced with lentiviral vectors expressing either GFP (MOI 5) or *IFI16* fused to monomeric GFP (MOI 5) in the presence of polybrene (8 µg/mL, Pierce, Rockford, IL, USA). After 72 hours, 70% confluent T-150 cell culture flasks were either mock-infected or infected with AAV2 (MOI 20,000), rAAVeGFP (MOI 20,000), or scAAVeGFP (MOI 20,000). At 24 hpi, the cells were washed once with ice-cold PBS, cross-linked with 1% formaldehyde in PBS, and incubated in a humidified, 95% air and 5% CO_2_ incubator at 37°C for 10 min. In order to stop the cross-linking, 125 mM glycine was added, and the cells were incubated for 5 min at RT. Next, the cells were washed twice with PBS, harvested using a cell scraper, and transferred into a 15 mL conical tube while being kept on ice. Next, the cells were pelleted at 1,000 *g* for 10 min at 4°C. The remaining pellet was resuspended in 50 µL of PBS and 100 µL of 1× SDS lysis buffer [100 mM Tris-HCl, pH 8.1, 1% SDS (wt/vol), 20 mM EDTA] containing protease inhibitor (cOmplete mini, Roche Cat. 11836153001). After 15 min of incubation on ice, the samples were centrifuged again, sonicated (100% amplitude, 15 s on, 15 s off, 20 min total sonication time), and the lysate was diluted 1:10 in dilution buffer [16.7 mM Tris-HCl, pH 8.1, 167 mM NaCl, 0.01% SDS (wt/vol), 1.2 mM EDTA, 1.1% Triton X-100 (vol/vol)]. Next, 80 µL of agarose protein G with salmon sperm DNA slurry (Millipore Cat. 16-201) was added for pre-clearance, and the samples were incubated with gentle agitation at 4°C for 30 min. The slurry was then centrifuged for 3 min at 4°C at 300 *g* (to not break the agarose beads), and the supernatant was recovered and split into two equal fractions. The fractions were then incubated either with 1.5 µg of anti-Sp1 antibody or rabbit IgG1 antibody at 4°C overnight with gentle agitation. The next day, 120 µL of agarose protein G with salmon sperm DNA slurry was added and incubated for 30 min at 4°C. The centrifugation for the following washing steps was performed at 4°C at 300 *g* for 3 min each. First, the samples were washed twice with low salt wash buffer [20 mM Tris-HCl, pH 8.1, 150 mM NaCl, 0.1% SDS (wt/vol), 2 mM EDTA, and 1% Triton X-100 (vol/vol)] and then with high salt wash buffer [20 mM Tris-HCl, pH 8.1, 500 mM NaCl, 0.1% SDS (wt/vol), 2 mM EDTA, 1% Triton X-100 (vol/vol)]. Next, the samples were washed with LiCl salt wash buffer [20 mM Tris-HCl, pH 8.1, 250 mM LiCl, 1% deoxycholate (wt/vol), 1 mM EDTA, and 1% Nonidet P-40 (vol/vol)] and finally twice with TE (pH 8.0). After centrifugation, the supernatant was discarded, and the samples were eluted twice in 250 µL of freshly prepared elution buffer [1% SDS (wt/vol) and 100 mM NaHCO_3_] for 15 min at 65°C, and eluates were combined. Next, 20 µL of 5 M NaCl was added to the samples, and they were incubated at 65°C overnight in order to reverse the cross-link. To recover the DNA, 10 µL of 0.5 M EDTA, 20 µL of 1 M Tris-HCl (pH 6.5), 2 µL of proteinase K (10 mg/mL), and 1 µL of RNase A (10 mg/mL) were added, and the samples were incubated at 45°C for 1 h. For phenol/chloroform extraction of the DNA, 1 volume of phenol:chloroform:isoamylalcohol (25:24:1, vol/vol, 15593031, Invitrogen, USA) was added. The samples were centrifuged for 5 min at 4°C and 15,500 *g*. The supernatant was transferred into a fresh tube, and 1 volume of chloroform was added. The sample was centrifuged for 1 min at 4°C and 15,500 *g*, and the supernatant was transferred into a fresh tube. 2.5 volumes of EtOH (pure) and 0.1 vol of 3 M NaAc (pH 5.5) were added to the sample. To precipitate the DNA, the suspension was incubated for at least 20 min at −80°C. Next, the samples were centrifuged for 10 min at 4°C and 18,000 *g*, and the supernatant was discarded. The DNA pellet was washed with 70% EtOH and centrifuged for 10 min at 4°C and 18,000 *g*. The supernatant was removed, and the DNA pellet was left to dry for at least 20 min at RT. After drying, the pellet was resuspended in Tris-HCl (pH 8.5) and incubated for 10 min at 37°C. For qPCR, 4 µL of the isolated DNA (approximately 10 ng) was used, and the rest was stored at −20°C. For each reaction, the following mixture was prepared: 1 µL of forward primer (10 µM), 1 µL of reverse primer (10 µM), 10 µL of SYBR Green PCR master mix, and 4 µL of ddH_2_O and transferred into a well of a Hard-Shell 96-well PCR plate (MicroAmp fast 96-well reaction plate). Four microliters of the appropriate DNA was added, and the 96-well plate was centrifuged for 1 min at 1,000 × *g* and subsequently run at the standard 20 µL qPCR SYBR green program on QuantStudio 3 real-time system (Applied Biosystem, ThermoFisher Scientific, Waltham, MA, USA). The experiment was performed as technical triplicates for each primer pair for each sample. The primer sequences used are listed in Table 4.

**TABLE 4:** ChIP primers used in this study

**Gene**	**Primers**
AAV p5	5′-ACCATGTGGTCACGCTGGG′ (forward) (53)
	5′-AAACCTCCCGCTTCAAAATGGA-3′ (reverse) (53)
AAV2 p19	5′-AGCGCCTGTTTGAATCTCACG-3′ (forward)
	5′-CTCTGCGTCTGCGACA-3′ (reverse)
CMV	5′-ATGACCTTATGGGACTTTCCTACTTGG-3′ (forward)
	5′-CCCGTGAGTCAAACCGCTATCC-3′ (reverse)

### Cell cycle analysis

NHF cells were seeded onto 6-well tissue culture plates at a confluency of 30% per plate and 24 h later infected with AAV2 or rAAVeGFP (MOI 5,000) in DMEM (0% FBS and 1% AB; pre-cooled to 4°C). The virus was allowed to adsorb at 4°C for 30 min before cultures were placed for 1 h into a humidified incubator at 37°C in a 95% air-5% CO_2_ atmosphere. After washing the cells with PBS and adding fresh medium (DMEM supplemented with 2% FBS and 1% AB), the cells were placed back at 37°C in a 95% air-5% CO_2_ atmosphere. At the indicated time points, the cells were harvested by exposing them to 0.05% Trypsin-EDTA solution for 10 min, centrifuged and washed with PBS, fixed in 2.5 mL ice-cold 100% ethanol, and stored overnight at −20°C. At the time of analysis, the cells were centrifuged, washed once again with PBS, and stained with a freshly made solution containing 0.1 mg/mL propidium iodide, 0.05% Triton X-100, and 0.1 mg/mL ribonuclease A (RNase A) in PBS. All samples were incubated for 40 min at 37°C in the dark. Cell cycle distribution was determined by flow cytometry (Gallios flow cytometer; Beckman Coulter, Brea, CA, USA), and data were analyzed by using Kaluza Flow Analysis software (Beckman Coulter, Brea, CA, USA).

### RNA interference

6 × 10^4^ NHF cells per well were plated in 24-well tissue culture plates and transfected using lipofectamine RNAiMax transfection reagent (ThermoFisher Scientific, Waltham, MA, USA) according to the manufacturer’s recommendations. The sequences of the siRNAs specific for *IFI16* (pool, 5′-UTR and cds), C911 siRNA controls, and siRNA targeting the cds of STING are listed in Table 5. At 40 hpt, the cells were transduced with recombinant AAV2 vectors as indicated in the Results and figure legends. Knockdown efficiency was assessed either by Western blotting or RT-qPCR.

**TABLE 5:** siRNAs used in this study

Target	Sequence or distributor
scr control	Control siRNA-A: sc-37007, Santa Cruz Biotechnology
IFI16 pool	IFI-16 siRNA (h): sc-35633, Santa Cruz Biotechnology
IFI16 5′-UTR	Hs_IFI16_7: SI04341092, Qiagen
IFI16 cds	Hs_IFI16_6: SI04156005, Qiagen
IFI16.2	5′-UCAGAAGACCACAAUCUACdTdT-3′ (54)
C911 IFI16.2	5′-UCAGAAGAGGUCAAUCUACdTdT-3′ (31)
IFI16.3	5′-ACACCAGCUUGAAGGAGAAdTdT-3′ (55)
C911 IFI16.3	5′-ACACCAGCAACAAGGAGAAdTdT-3′ (31)
STING	Hs_TMEM173_3: SI04287626, Qiagen
GFP control	Ctrl_GFP_2: SI04380467, Qiagen

### Exogenous complementation

6 × 10^4^ U2OS IFI16^-/-^ were either untransduced, transduced with lentiviral vectors expressing GFP (MOI 5), or lentiviral vectors expressing *IFI16* fused to monomeric GFP (MOI 5) in the presence of polybrene (8 µg/mL, Pierce, Rockford, IL, USA). After 72 hours, the cells were infected with rAAV2mCherry (MOI 500), and 24 hpi, mCherry and *IFI16* expression was assessed by RT-qPCR using specific primers for mCherry or *IFI16* (Table 1), respectively.

### Microscopy

NHF cells were seeded onto coverslips (12 mm diameter; Glaswarenfabrik Karl Hecht GmbH & Co. KG, Sondheim, Germany) in 24-well tissue culture plates (4 × 10^4^ cells per well). The next day, the cells were infected as indicated in the Results and the figure legends. For immunofluorescence analysis and CSLM, the cells were washed once with cold PBS 24 h after infection and then fixed with 2% paraformaldehyde (PFA) in PBS for 10 min at RT. The fixation process was stopped by incubation with 0.1 M glycine for 10 min at RT and two washes with cold PBS. Afterward, the cells were permeabilized with 0.1% Triton-X 100 (in PBS) for 10 min, followed by three washing steps with PBS. The cells were blocked for 30 min with 3% bovine serum albumin (BSA) in PBS-T (0.05% Tween) at 4°C. For staining, the cells were incubated with antibodies diluted in PBS-T-BSA (3%) in a humidified chamber at RT in the dark. The coverslips were placed onto droplets (20 µL) of a primary or secondary antibody solution. After incubation for 1 h, the cells were washed three times with PBS and once with H_2_O. All coverslips were embedded in ProLong Anti-Fade mountant (Molecular Probes), and cells were observed by using a confocal laser scanning microscope (Leica SP8; Leica Microsystems, Wetzlar, Germany). To prevent crosstalk between the channels for the different fluorochromes, all channels were recorded separately, and fluorochromes with longer wavelengths were recorded first.

### Fluorescence *in situ* hybridization

FISH was performed essentially as described previously by Lux et al. (56). Briefly, a 3.9-kb DNA fragment containing the AAV2 genome without the inverted terminal repeats was amplified by PCR from plasmid pDG using forward (5′-CGGGGTTTTACGAGATTGTG-3′) and reverse (5′-GGCTCTGAATACACGCCATT-3′) primers and the following conditions: 30 s at 95°C; 35 cycles of 10 s at 98°C, 15 s at 58°C, and 75 s at 72°C; and 10 min at 72°C. The PCR sample was then digested with DpnI to cut the residual template DNA and purified with the Pure Link PCR Purification Kit (Qiagen, Hilden, Germany). The DNA fragment was labeled with 5-(3-aminoallyl)dUTP by nick translation, and the incorporated dUTPs were labeled with amino-reactive Alexa Fluor 647 dye by using the Ares DNA labeling kit (Molecular Probes, Eugene, OR, USA) according to the manufacturer’s protocols. NHF cells were plated onto glass coverslips in 24-well plates at a density of 4 × 10^4^ cells per well, and 24 h later, the cells were mock infected or infected with AAV2 (MOI of 20,000). Twenty-four hours after infection, the cells were washed with PBS, fixed for 30 min at RT with 2% PFA (in PBS), and then washed again with PBS. The cells were then quenched for 10 min with 50 mM NH_4_Cl (in PBS), washed with PBS, permeabilized for 10 min with 0.2% Triton X-100 (in PBS), blocked for 10 min with 0.2% gelatin (in PBS), and washed again with PBS. Hybridization solution (20 µL per coverslip) containing 1 ng/µL of the labeled DNA probe, 50% formamide, 7.3% dextran sulfate, 15 ng/µL salmon sperm DNA, and 0.74× SSC (1× SSC is 0.15 M NaCl plus 0.015 M sodium citrate) was denatured for 3 min at 95°C and shock-cooled on ice. The coverslips with the fixed and permeabilized cells facing down were placed onto a drop (20 µL) of the denatured hybridization solution and incubated overnight at 37°C in a humidified chamber (note that the cells were not denatured, as the AAV2 genome is present as ssDNA). The next day, the coverslips were washed three times with 2× SSC at 37°C, three times with 0.1× SSC at 60°C, and twice with PBS at RT. The cells were then embedded in ProLong Anti-Fade mountant (Molecular Probes, Eugene, OR, USA) and imaged by confocal laser scanning microscopy (Leica SP8; Leica Microsystems, Wetzlar, Germany).

## Data Availability

Sequencing data (full length and *P* < 0.01, number of reads > 40) are available under Zenodo (https://doi.org/10.5281/zenodo.7147541).

## SUPPLEMENTAL MATERIAL

Fig. S1 jvi.00110-24-s0001.eps Selected heat maps of affected biological processes.

Fig. S2 jvi.00110-24-s0002.eps Clustered heat map of 872 genes.

Fig. S3 jvi.00110-24-s0003.eps Cell cycle phase distributions upon AAV2 or rAAV2 infection over time.

Fig. S4 jvi.00110-24-s0004.eps C911 siRNA controls.

Fig. S5 jvi.00110-24-s0005.eps STING signaling in different cell lines.

Fig. S6 jvi.00110-24-s0006.eps Nucleolar localization of IFI16.

Supplemental legends jvi.00110-24-s0007.docx Legends for Fig. S1 to S6 and Tables S1 and S2.

Table S1 jvi.00110-24-s0008.docx List of the enrichment map of GO terms according to Fig. 1.

Table S2 jvi.00110-24-s0009.txt List of all genes in the different GO terms according to Fig. 1.

ASM does not own the copyrights to Supplemental Material that may be linked to, or accessed through, an article. The authors have granted ASM a non-exclusive, world-wide license to publish the Supplemental Material files. Please contact the corresponding author directly for reuse.
