# Supplementary material for: Interferon-γ inducible factor 16 (IFI16) restricts adeno-associated virus type 2 (AAV2) transduction in an immune-modulatory independent way
Source: J Virol. 2024 Jun 5;98(7):e00110-24. doi: 10.1128/jvi.00110-24 (PMC11338077; doi:10.1128/jvi.00110-24)
Supplement: Supplemental legends — Legends for Fig. S1 to S6 and Tables S1 and S2. [file jvi.00110-24-s0007.docx]

**Legends Supplementary Figures**

**FIG S1** Selected heat maps of affected biological processes. Reads of the 50 most differentially expressed genes (depicted on the right of each heat map) between AAV2-infected cells (A1-A3) and mock-infected cells (M1-M3) from (A) the cell cycle and (B) chromatin organization clusters of the enrichment map. The dendrogram (left side of each heat map) illustrates the unsupervised clustering of the genes.

**FIG S2** Clustered heat map of 872 genes with a log_2_ ratio > |0.58| and a significance threshold of p < 0.01 were used for the unsupervised clustering of the genes. AAV2-infected samples (A1-A3) are represented on the left and mock-infected samples (M1-M3) on the right.

**FIG S3** Cell cycle phase distributions upon AAV2 or rAAV2 infection over time.

NHF cells were infected with AAV2 or rAAVeGFP (MOI 5`000). At the indicated time points, the cell cycle profile was assessed by PI staining and flow cytometry (10`000 cells per sample). Graph shows mean and SD of the percentage of cells in each cell cycle phase at the individual time points. p-values were calculated using an unpaired Student`s t-test (* - p ≤ 0.05, ** - p ≤ 0.01, *** - p ≤ 0.001, **** - p ≤ 0.0001).

**FIG S4** C911 siRNA controls. NHF cells were reverse transfected with no siRNA, scr control siRNA or siRNAs targeting the coding sequence of *IFI16* (IFI16 pool, IFI16.2 and IFI16.3). To address the question of off-target effects of the individual siRNAs (IFI16.2, IFI16.3), C911 siRNA controls were included. 36 hpt the cells were either mock-infected or infected with rAAVeGFP (MOI 4`000). (A) 24 hpi, cells were counted using a fluorescence microscope. (B) The graph shows mean and SD of the relative cell count of GFP positive NHF cells from triplicate experiments. p-values were calculated using an unpaired Student`s t-test (* - p ≤ 0.05, ** - p ≤ 0.01, *** - p ≤ 0.001, **** - p ≤ 0.0001). (C) Knock-down of *IFI16* was confirmed on protein level.

**FIG S5** STING signaling in different cell lines. NHF, U2OS and HeLa cells were treated with 2’3’-cGAMP (3 μM) for 9 h, and total RNA was extracted, converted to cDNA and subjected to RT-qPCR using specific primers for *STING* and *ISG56*.

p-values were calculated using an unpaired Student`s t-test (* - p ≤ 0.05, ** - p ≤ 0.01, *** - p ≤ 0.001, **** - p ≤ 0.0001).

**FIG S6** Nucleolar localization of IFI16. NHF cells were infected with AAV2 (MOI 20`000). After 24 h, the cells were fixed and processed for multicolor IF and CLSM. IFI16 was detected by using a monoclonal antibody against IFI16 (green/blue). Nucleoli were visualized using an antibody against fibrillarin (red). Nuclei were counterstained with DAPI.

**Legends Supplementary Tables**

Supplementary Table 1 List of the enrichment map of GOterms according to Fig. 1. NodeName represents the individual GOterms, while GSSize corresponds to the number of genes included. For each NodeName p-values (pVal) and false discovery rate (FDR) are indicated. The most affected biological processes are summarized as keywords (in bold).

Supplementary Table 2 List of all genes in the different GOterms according to Fig. 1. NodeName represents the individual GOterms, while GenSetSize corresponds to the number of genes included. For each NodeName, GenSetSize, p-values (pVal), false discovery rate (FDR), and genes included, are listed.
