# Supplementary material for: Interferon-γ inducible factor 16 (IFI16) restricts adeno-associated virus type 2 (AAV2) transduction in an immune-modulatory independent way
Source: J Virol. 2024 Jun 5;98(7):e00110-24. doi: 10.1128/jvi.00110-24 (PMC11338077; doi:10.1128/jvi.00110-24)
Supplement: Table S1 — List of the enrichment map of GO terms according to Fig. 1. [file jvi.00110-24-s0008.docx]

| **NodeName** | **GSSize** | **pVal** | **FDR** |
| --- | --- | --- | --- |
| **Regulation of macromolecules /Metabolic Processes / Gene expression** | | | |
| ANAPHASE-PROMOTING COMPLEX-DEPENDENT PROTEASOMAL UBIQUITIN-DEPENDENT PROTEIN CATABOLIC PROCESS | 19 | 3.750E-05 | 1.669E-03 |
| CELLULAR PROTEIN CATABOLIC PROCESS | 92 | 6.060E-05 | 2.566E-03 |
| MODIFICATION-DEPENDENT MACROMOLECULE CATABOLIC PROCESS | 87 | 1.290E-04 | 4.903E-03 |
| MODIFICATION-DEPENDENT PROTEIN CATABOLIC PROCESS | 87 | 1.290E-04 | 4.903E-03 |
| NEGATIVE REGULATION OF CATALYTIC ACTIVITY | 50 | 7.290E-05 | 2.983E-03 |
| NEGATIVE REGULATION OF CELLULAR PROTEIN METABOLIC PROCESS | 48 | 8.840E-10 | 1.290E-07 |
| NEGATIVE REGULATION OF LIGASE ACTIVITY | 18 | 1.990E-04 | 6.774E-03 |
| NEGATIVE REGULATION OF MACROMOLECULE METABOLIC PROCESS | 116 | 4.400E-07 | 4.010E-05 |
| NEGATIVE REGULATION OF MOLECULAR FUNCTION | 57 | 9.290E-05 | 3.639E-03 |
| NEGATIVE REGULATION OF PROTEIN METABOLIC PROCESS | 49 | 1.050E-09 | 1.480E-07 |
| NEGATIVE REGULATION OF PROTEIN MODIFICATION PROCESS | 31 | 1.010E-06 | 7.830E-05 |
| NEGATIVE REGULATION OF UBIQUITIN-PROTEIN LIGASE ACTIVITY | 18 | 1.990E-04 | 6.774E-03 |
| NEGATIVE REGULATION OF UBIQUITIN-PROTEIN LIGASE ACTIVITY DURING MITOTIC CELL CYCLE | 18 | 1.340E-04 | 4.958E-03 |
| POSITIVE REGULATION OF CELLULAR PROTEIN METABOLIC PROCESS | 46 | 1.720E-05 | 8.600E-04 |
| POSITIVE REGULATION OF LIGASE ACTIVITY | 21 | 1.680E-05 | 8.510E-04 |
| POSITIVE REGULATION OF PROTEIN METABOLIC PROCESS | 48 | 1.050E-05 | 5.450E-04 |
| POSITIVE REGULATION OF PROTEIN MODIFICATION PROCESS | 37 | 1.450E-04 | 5.233E-03 |
| POSITIVE REGULATION OF PROTEIN UBIQUITINATION | 21 | 1.460E-04 | 5.223E-03 |
| POSITIVE REGULATION OF UBIQUITIN-PROTEIN LIGASE ACTIVITY | 20 | 3.130E-05 | 1.428E-03 |
| POSITIVE REGULATION OF UBIQUITIN-PROTEIN LIGASE ACTIVITY DURING MITOTIC CELL CYCLE | 20 | 2.000E-05 | 9.740E-04 |
| POSTTRANSCRIPTIONAL REGULATION OF GENE EXPRESSION | 45 | 1.160E-06 | 8.630E-05 |
| PROTEASOMAL PROTEIN CATABOLIC PROCESS | 27 | 4.190E-06 | 2.510E-04 |
| PROTEASOMAL UBIQUITIN-DEPENDENT PROTEIN CATABOLIC PROCESS | 27 | 4.190E-06 | 2.510E-04 |
| PROTEIN CATABOLIC PROCESS | 94 | 7.040E-05 | 2.915E-03 |
| PROTEOLYSIS INVOLVED IN CELLULAR PROTEIN CATABOLIC PROCESS | 92 | 5.030E-05 | 2.156E-03 |
| REGULATION OF CELLULAR PROTEIN METABOLIC PROCESS | 98 | 3.370E-12 | 6.460E-10 |
| REGULATION OF LIGASE ACTIVITY | 24 | 2.040E-06 | 1.430E-04 |
| REGULATION OF PHOSPHATE METABOLIC PROCESS | 74 | 2.510E-04 | 8.305E-03 |
| REGULATION OF PHOSPHORUS METABOLIC PROCESS | 74 | 2.510E-04 | 8.305E-03 |
| REGULATION OF PROTEIN MODIFICATION PROCESS | 56 | 5.220E-06 | 3.070E-04 |
| REGULATION OF PROTEIN UBIQUITINATION | 24 | 8.520E-05 | 3.410E-03 |
| REGULATION OF TRANSFERASE ACTIVITY | 60 | 2.790E-04 | 9.045E-03 |
| REGULATION OF TRANSLATION | 34 | 9.050E-07 | 7.180E-05 |
| REGULATION OF TRANSLATIONAL INITIATION | 17 | 6.830E-07 | 5.670E-05 |
| REGULATION OF UBIQUITIN-PROTEIN LIGASE ACTIVITY | 23 | 3.830E-06 | 2.410E-04 |
| REGULATION OF UBIQUITIN-PROTEIN LIGASE ACTIVITY DURING MITOTIC CELL CYCLE | 22 | 2.740E-06 | 1.820E-04 |
| TRANSLATION | 55 | 2.580E-04 | 8.440E-03 |
| UBIQUITIN-DEPENDENT PROTEIN CATABOLIC PROCESS | 48 | 4.100E-06 | 2.490E-04 |
| **Cell cycle regulation** | | | |
| CELL CYCLE | 168 | 2.500E-23 | 4.570E-20 |
| CELL CYCLE CHECKPOINT | 31 | 1.220E-09 | 1.650E-07 |
| CELL CYCLE PHASE | 103 | 1.370E-18 | 1.000E-15 |
| CELL CYCLE PROCESS | 131 | 8.850E-21 | 1.080E-17 |
| CELL DIVISION | 74 | 5.760E-14 | 1.310E-11 |
| CHROMOSOME SEGREGATION | 26 | 1.260E-07 | 1.240E-05 |
| CYTOKINESIS | 16 | 3.960E-06 | 2.450E-04 |
| DNA INTEGRITY CHECKPOINT | 17 | 2.410E-05 | 1.127E-03 |
| ESTABLISHMENT OF ORGANELLE LOCALIZATION | 21 | 6.640E-06 | 3.720E-04 |
| INTERPHASE | 29 | 8.340E-07 | 6.760E-05 |
| INTERPHASE OF MITOTIC CELL CYCLE | 29 | 4.400E-07 | 3.920E-05 |
| M PHASE | 84 | 1.020E-15 | 2.600E-13 |
| M PHASE OF MITOTIC CELL CYCLE | 68 | 1.780E-17 | 5.900E-15 |
| MICROTUBULE CYTOSKELETON ORGANIZATION | 36 | 5.740E-07 | 4.980E-05 |
| MICROTUBULE-BASED PROCESS | 53 | 2.050E-07 | 1.920E-05 |
| MITOSIS | 68 | 6.330E-18 | 2.570E-15 |
| MITOTIC CELL CYCLE | 107 | 2.760E-25 | 1.010E-21 |
| MITOTIC CELL CYCLE CHECKPOINT | 17 | 1.450E-06 | 1.060E-04 |
| MITOTIC SISTER CHROMATID SEGREGATION | 13 | 1.050E-04 | 4.080E-03 |
| NEGATIVE REGULATION OF CELL CYCLE | 21 | 8.530E-05 | 3.376E-03 |
| NUCLEAR DIVISION | 68 | 6.330E-18 | 2.570E-15 |
| ORGANELLE FISSION | 69 | 1.550E-17 | 5.670E-15 |
| ORGANELLE LOCALIZATION | 22 | 1.910E-04 | 6.615E-03 |
| REGULATION OF CELL CYCLE | 80 | 1.280E-13 | 2.750E-11 |
| REGULATION OF CELL CYCLE PROCESS | 33 | 1.070E-07 | 1.080E-05 |
| REGULATION OF MITOSIS | 17 | 2.460E-04 | 8.189E-03 |
| REGULATION OF MITOTIC CELL CYCLE | 45 | 9.660E-11 | 1.680E-08 |
| REGULATION OF MITOTIC METAPHASE/ANAPHASE TRANSITION | 10 | 1.290E-04 | 4.943E-03 |
| REGULATION OF NUCLEAR DIVISION | 17 | 2.460E-04 | 8.189E-03 |
| SISTER CHROMATID SEGREGATION | 13 | 1.420E-04 | 5.169E-03 |
| SPINDLE ORGANIZATION | 17 | 2.930E-06 | 1.870E-04 |
| **Chromatin organization** | | | |
| CELLULAR MACROMOLECULAR COMPLEX ASSEMBLY | 103 | 4.800E-10 | 7.620E-08 |
| CELLULAR MACROMOLECULAR COMPLEX SUBUNIT ORGANIZATION | 112 | 2.140E-11 | 3.900E-09 |
| CHROMATIN ASSEMBLY | 74 | 2.210E-17 | 6.720E-15 |
| CHROMATIN ASSEMBLY OR DISASSEMBLY | 85 | 6.220E-19 | 5.670E-16 |
| CHROMATIN ORGANIZATION | 119 | 4.660E-13 | 9.450E-11 |
| CHROMATIN REMODELING | 17 | 6.630E-05 | 2.777E-03 |
| CHROMOSOME ORGANIZATION | 147 | 1.480E-18 | 8.990E-16 |
| DNA PACKAGING | 82 | 2.690E-18 | 1.220E-15 |
| MACROMOLECULAR COMPLEX ASSEMBLY | 134 | 1.640E-05 | 8.440E-04 |
| MACROMOLECULAR COMPLEX SUBUNIT ORGANIZATION | 141 | 7.230E-06 | 4.000E-04 |
| NUCLEOSOME ASSEMBLY | 73 | 3.890E-17 | 1.090E-14 |
| NUCLEOSOME ORGANIZATION | 73 | 2.810E-15 | 6.750E-13 |
| PROTEIN-DNA COMPLEX ASSEMBLY | 76 | 2.670E-18 | 1.390E-15 |
| **Intracellular transport** | | | |
| CELL PROLIFERATION | 68 | 1.500E-04 | 5.246E-03 |
| CELLULAR MACROMOLECULE LOCALIZATION | 68 | 2.990E-05 | 1.378E-03 |
| CELLULAR PROTEIN LOCALIZATION | 66 | 7.960E-05 | 3.222E-03 |
| ESTABLISHMENT OF PROTEIN LOCALIZATION | 123 | 5.080E-08 | 5.790E-06 |
| GOLGI VESICLE TRANSPORT | 31 | 8.350E-06 | 4.410E-04 |
| INTRACELLULAR TRANSPORT | 104 | 1.050E-06 | 7.980E-05 |
| MAINTENANCE OF LOCATION IN CELL | 15 | 1.470E-04 | 5.192E-03 |
| MEMBRANE ORGANIZATION | 60 | 2.960E-04 | 9.442E-03 |
| PROTEIN LOCALIZATION | 141 | 4.590E-09 | 5.400E-07 |
| PROTEIN TRANSPORT | 122 | 5.480E-08 | 6.060E-06 |
| VESICLE-MEDIATED TRANSPORT | 97 | 1.440E-07 | 1.380E-05 |
| **Cytoskeleton / Organelle organization** | | | |
| ACTIN CYTOSKELETON ORGANIZATION | 45 | 7.730E-06 | 4.210E-04 |
| ACTIN FILAMENT-BASED PROCESS | 46 | 1.870E-05 | 9.200E-04 |
| CYTOSKELETON ORGANIZATION | 86 | 4.580E-10 | 7.590E-08 |
| NEGATIVE REGULATION OF CELLULAR COMPONENT ORGANIZATION | 37 | 7.380E-08 | 7.690E-06 |
| NEGATIVE REGULATION OF ORGANELLE ORGANIZATION | 22 | 3.190E-05 | 1.436E-03 |
| REGULATION OF CYTOSKELETON ORGANIZATION | 30 | 4.860E-05 | 2.107E-03 |
| REGULATION OF MICROTUBULE CYTOSKELETON ORGANIZATION | 14 | 1.290E-04 | 4.857E-03 |
| REGULATION OF ORGANELLE ORGANIZATION | 50 | 5.810E-08 | 6.240E-06 |
| **Cell motion** | | | |
| NEGATIVE REGULATION OF CELL MIGRATION | 16 | 3.030E-04 | 9.581E-03 |
| NEGATIVE REGULATION OF CELL MOTION | 19 | 2.360E-05 | 1.118E-03 |
| REGULATION OF CELL MIGRATION | 39 | 2.440E-06 | 1.650E-04 |
| REGULATION OF CELL MOTION | 44 | 6.170E-07 | 5.240E-05 |
| REGULATION OF LOCOMOTION | 40 | 2.150E-05 | 1.032E-03 |
| **DNA replication / DNA damage response** | | | |
| CELLULAR RESPONSE TO STRESS | 103 | 8.280E-10 | 1.260E-07 |
| DNA DAMAGE RESPONSE, SIGNAL TRANSDUCTION | 23 | 6.040E-06 | 3.490E-04 |
| DNA METABOLIC PROCESS | 93 | 3.670E-09 | 4.460E-07 |
| DNA REPAIR | 49 | 1.360E-04 | 5.012E-03 |
| DNA REPLICATION | 48 | 1.850E-09 | 2.410E-07 |
| DNA-DEPENDENT DNA REPLICATION | 19 | 6.620E-06 | 3.770E-04 |
| RESPONSE TO DNA DAMAGE STIMULUS | 75 | 2.850E-09 | 3.580E-07 |
| RESPONSE TO UV | 20 | 1.980E-06 | 1.420E-04 |
| **Apoptosis** | |  |  |
| CELL DEATH | 102 | 2.240E-04 | 7.532E-03 |
| DEATH | 102 | 2.800E-04 | 9.006E-03 |
| REGULATION OF ANTI-APOPTOSIS | 14 | 4.000E-05 | 1.754E-03 |
| REGULATION OF APOPTOSIS | 119 | 8.050E-06 | 4.320E-04 |
| REGULATION OF CELL DEATH | 123 | 2.090E-06 | 1.440E-04 |
| REGULATION OF PROGRAMMED CELL DEATH | 122 | 2.910E-06 | 1.900E-04 |

**Supplementary Table 1** List of the enrichment map of GOterms according to Fig. 1. NodeName represents the individual GOterms, while GSSize corresponds to the number of genes included. For each NodeName p-values (pVal) and false discovery rate (FDR) are indicated. The most affected biological processes are summarized as keywords (in bold).
